# Slab underthrusting is the primary control on flat-slab size

**DOI:** 10.1126/sciadv.adv8872

**Published:** 2025-07-11

**Authors:** Guido M. Gianni, Leandro C. Gallo, Jeremías Likerman, Andrés Echaurren, Conrado R. Gianni, Claudio Faccenna

**Affiliations:** ^1^ German Research Centre for Geosciences (GFZ), Potsdam 14473, Germany.; ^2^ Centre for Planetary Habitability, University of Oslo, Oslo 0316, Norway.; ^3^ National Scientific and Technical Research Council (CONICET), Capital Federal 1414, Argentina.; ^4^ Dip. Scienze, Università degli Studi Roma Trè, Rome 00154, Italy.

## Abstract

Flat subduction, an intermittent phenomenon along active margins, arises from well-known causes, yet the mechanisms driving its expansion remain poorly understood. The prevailing view suggests that trenchward continental motion drives slab overthrusting, causing the flat slab to expand oceanward. Here, we explore an alternative mechanism: underthrusting of the subducting plate through forward propagation of the flat-slab hinge. We directly evaluate both hypotheses through a kinematic analysis of trench and flat-slab motions using a global flat subduction database cast into multiple absolute plate motion models. Our results indicate that flat-slab expansion reflects distinct end-member processes, with forward propagation emerging as the dominant mode. We present a framework for flat-slab propagation that emphasizes the dynamic interaction between lower-plate motion and slab pull from adjacent subduction zones, an interplay validated through numerical modeling. These findings challenge conventional assumptions and underscore the need to reconsider the role of lower-plate kinematics in flat-slab dynamics.

## Teaser

Flat-slab expansion is driven primarily by underthrusting of the lower plate, challenging long-standing geodynamic views.

## INTRODUCTION

Flat subduction, characterized by slab dipping angles of ≤10° (*1*, *2*), represents a distinctive geodynamic phenomenon with far-reaching implications. It influences magmatic activity, crustal deformation, seismicity frequency, basin subsidence, and lithospheric thermal state in subduction zones (*3*–*6*). Now, flat subduction manifests in discrete segments of the circum-Pacific Ocean subduction (*5*) and has been extensively documented in ancient active margins (*3*, *7*–*15*). Conceptual and numerical modeling studies have devoted efforts to decipher the driving mechanisms and geological consequences associated with this phenomenon. While these studies have advanced our understanding of the causes of slab flattening by highlighting the roles of specific properties, structure, and kinematics of the lower and/or upper plates [e.g., (*10*, *11*, *16*–*21*)], the mechanisms driving the pronounced horizontal expansion of subduction zones after achieving a flat geometry remain poorly understood.

**Fig. 1.**
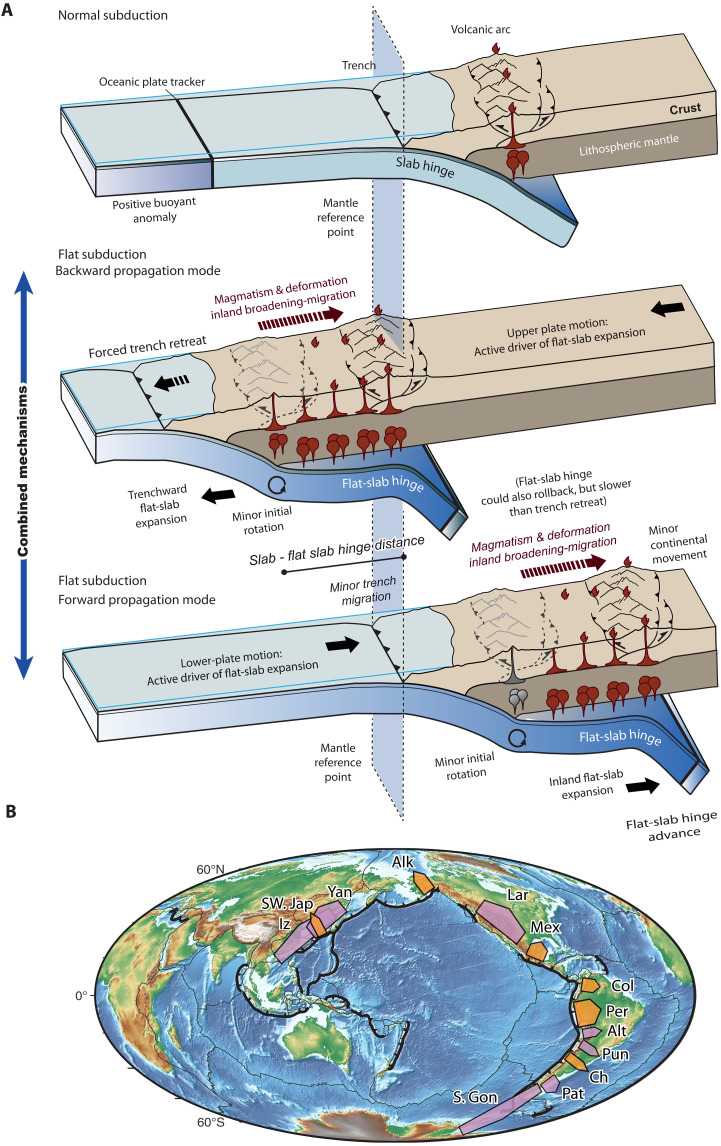
Conceptual model for end-member mechanisms for flat subduction expansion and location of active and ancient flat subduction settings. (**A**) The backward propagation mode is marked by an oceanward expansion of flat subduction, opposing the migration of arc magmatism and upper-plate compression. In contrast, forward propagation of flat subduction involves an inland expansion aligned with the migration directions of arc magmatism and upper-plate compression. The depiction of a stationary sublithospheric slab hinge at the leading edge of the flat slab in the former case is simplified as it is only one of the possibilities. (**B**) Global map showing the location of active (*5*, *14*) and ancient (*7*–*11*, *13*, *14*, *24*, *35*) flat-slab events analyzed in this study. Active and ancient flat slabs are shown in orange and purple labels, respectively. SW. Jap, Southwest Japan; Alk, Alaska; Mex, Mexico; Col, Colombia; Per, Peru; Ch, Chile; Lar, Laramide; Yan, Yanshanian; Iz, Izanagi; Pun, Puna; Alt, Altiplano; Pat, Patagonian Nalé; S. Gon, South Gondwana flat slabs. See details in data S1.

The formation and propagation of flat slabs have traditionally been regarded as driven by the same mechanism, overlooking the possibility that they are distinct yet interconnected processes {i.e., the flat slab is first formed by any of the suggested causes [e.g., (*10*, *11*, *16*–*21*)] and then continues propagating horizontally during plate convergence}. This lack of distinction has led some studies to support the early notion of large-scale slab angle changes (*3*) as the primary mechanism controlling the final extent of flat slabs [e.g., (*11*, *22*)]. However, changes in slab dip involving extensive slab segments, such as those observed in the broad flat slabs of Peru and Alaska [~400 to 460 km; (*5*, *23*)] or in the extinct Late Cretaceous-Paleogene Laramide flat slab [~1200 to 1500 km; (*24*)], are not replicated in subduction models, as they would require the displacement of a large volume of mantle. Instead, these models show a change in geometry mostly at flat-slab initiation and simulate oceanward or landward propagation during plate convergence [e.g., (*16*, *18*, *25*)]. Recent experimental (*16*–*19*) and kinematic (*26*) studies in active settings in central Chile and Peru suggest that the horizontal propagation of flat slabs most likely results from oceanward expansion driven by the trenchward motion of the advancing upper plate relative to the mantle (i.e., continental overthrusting), supporting the early hypothesis proposed by Barazangi and Isacks in the 1970s (*27*). In this mode, flat slabs propagate when the total amount of trench retreat exceeds the retreat of the slab hinge (*26*) (i.e., slab rollback; see Fig. 1A). We refer to this mechanism (*16*–*19*, *26*, *27*) as the backward-propagation mode.

Here, we examine the motion of the slab hinge relative to the mantle to assess a natural alternative: That flat slabs may expand inland beneath the continent due to forward migration of the slab hinge (i.e., flat-slab hinge advance and slab underthrusting). We refer to this mechanism as the forward-propagation mode (Fig. 1A). Although this mechanism has not yet been demonstrated to operate in natural settings (*16*–*18*, *26*), numerical experiments have replicated this behavior, suggesting that forward propagation is a geodynamically viable process (*10*, *24*, *25*). Thus, identifying the most viable propagation mechanism in natural settings and developing robust methods for its assessment are crucial for advancing our understanding of flat-slab dynamics. A formal conceptualization and a clear distinction of the mechanisms responsible for the propagation of flat subduction have yet to be established.

Here, we address this issue by integrating calculations of trench and flat-slab hinge kinematics and flat-slab size across both active and extinct flat-slab events (Fig. 1B). To achieve this, we analyzed well-resolved absolute plate motion models (*28*–*33*), which were compared with a global subduction geometry model (*34*) and space-time magmatic patterns, providing insights into the extents of both active and ancient flat slabs. Last, we complement these findings with two-dimensional (2D) geodynamic modeling of flat-slab propagation, enabling us to evaluate the underlying dynamics of this enigmatic process.

## RESULTS

### Unraveling flat subduction propagation modes

**Fig. 2.**
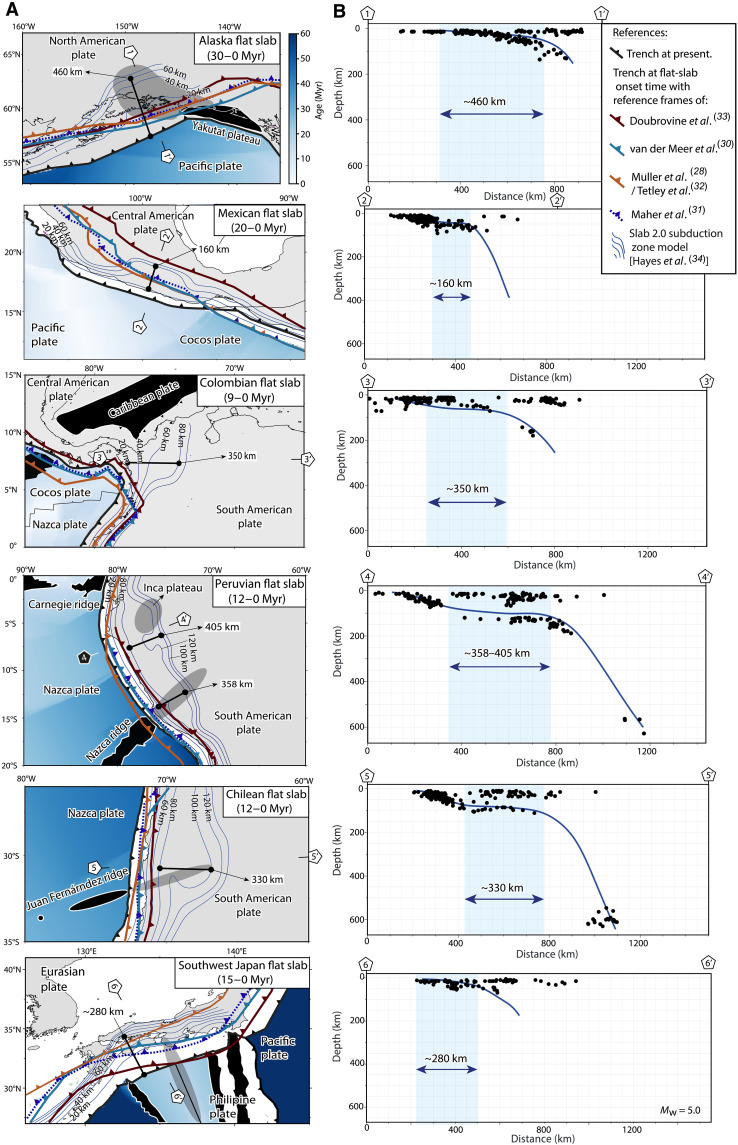
Comparison between present and reconstructed absolute trench position at the time of active flat-slab initiation and analysis of flat-slab extents. (**A**) Plate reconstructions to obtain absolute trench motion since the onset of each active flat slab. (**B**) Analysis of flat-slab extents from the Slab 2.0 model (*34*). This analysis was complemented with an inspection of a compilation of regional seismological surveys (see fig. S1). *M*_w_, moment magnitude.

**Fig. 3.**
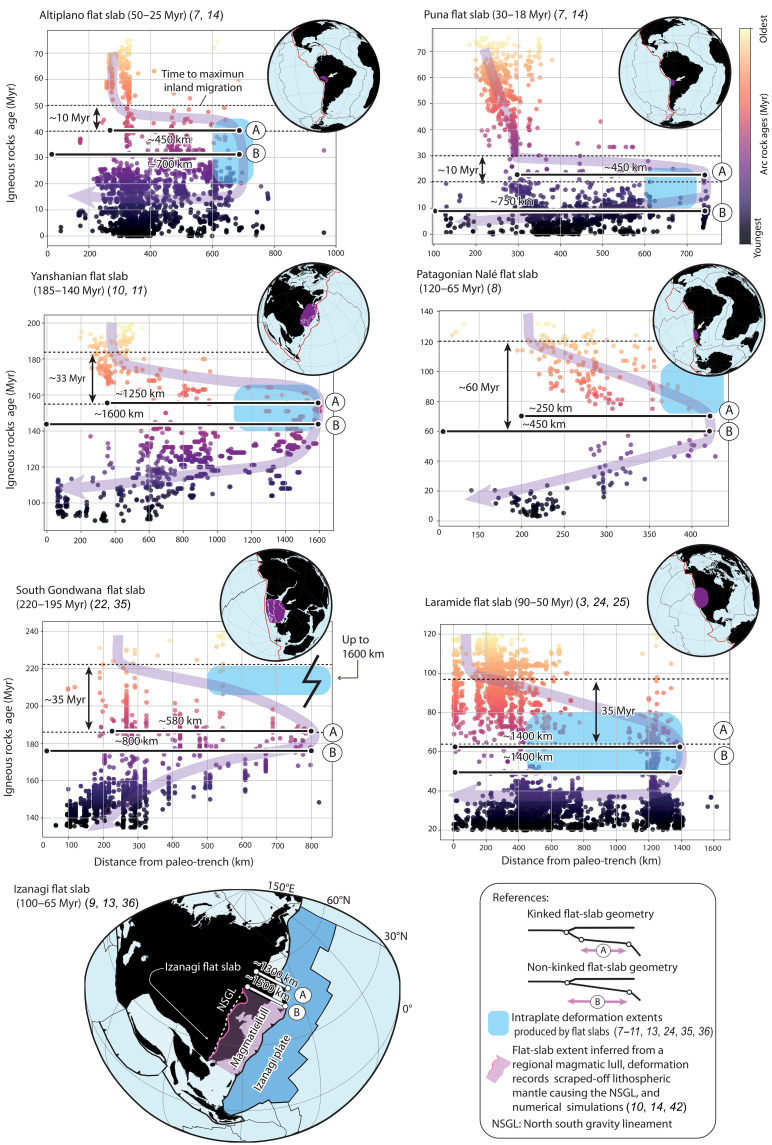
Spatiotemporal analysis of subduction-related igneous rocks for ancient flat subduction events. The analysis uses the time-space evolution of igneous rocks (*3*, *4*) to obtain potential flat-slab extents and time of flat subduction development as derived from time to maximum inland arc migration (study cases details are provided in data S1). Bars labeled as A and B correspond to estimates of flat-slab extents based on assuming kinked and non-kinked flat subduction geometries, respectively (see Results).

To better understand the mechanisms of flat subduction propagation, we performed a global analysis of modern and ancient flat slabs (Fig. 1B and data S1) (*5*, *7*–*10*, *13*, *14*, *24*, *35*, *36*). Similarly to the global analysis of Gutscher *et al.* (*5*), we combined subhorizontal and low-angle subduction, as both exhibit similar tectonic, magmatic, and seismotectonic characteristics show very shallow slab dip angles (≤10°) (*14*) and differ only in subtle geometric features, such as the presence of an additional slab kink in the latter (Figs. 2 and 3).

**Fig. 4.**
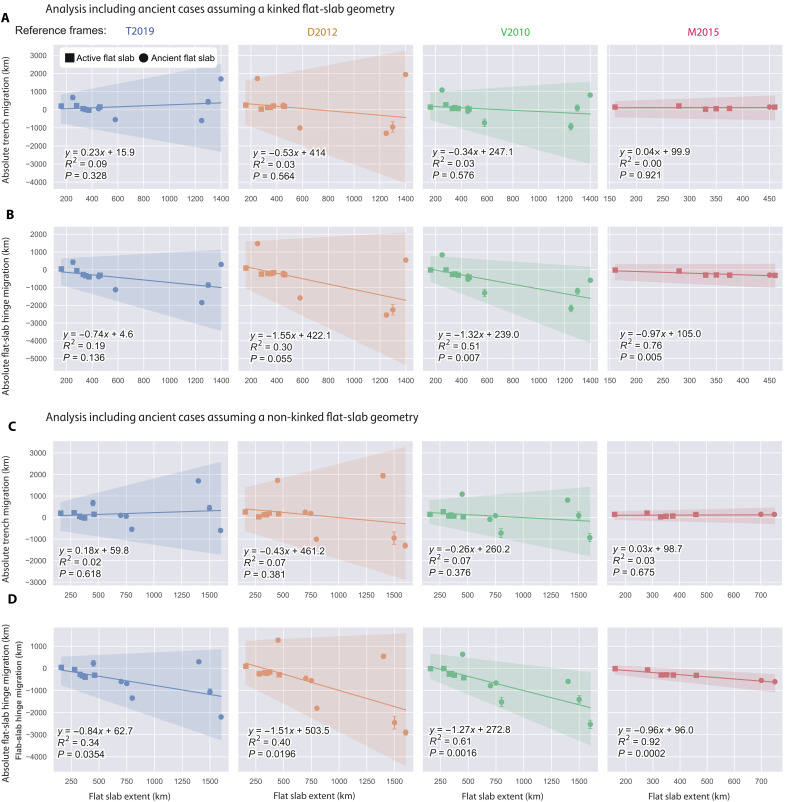
Statistical analysis of the absolute trench and flat-slab hinge motion versus flat subduction extents for active and ancient cases. Correlation analysis between (**A**) determined absolute trench and (**B**) flat-slab hinge motion versus flat-slab extents derived from plate kinematic models (*28*, *29*, *37*, *38*) with different mantle reference frames (*30*–*33*). Both (A) and (B) consider ancient flat-slab extents determined assuming a kinked flat-slab geometry. (**C** and **D**) Equal to (A) and (B), respectively, but assuming a non-kinked flat-slab geometry in the determination of flat-slab extent for ancient cases (see details in data S1). The diagrams display a bootstrapped linear regression model and associated 95% error margins, derived from 1000 bootstrap samples. Uncertainty in the extent of flat slab is incorporated through Monte Carlo sampling, considering error distributions with a SD. Please also note that, due to limitations inherent in certain reference frames, certain flat slabs may lack absolute trench motion data (see further explanation in Materials and Methods).

We performed an analysis, shown in Fig. 4 (A to D), that integrates the calculated inland extents of both active and ancient flat slabs with the dynamics of the underlying mantle (see Materials and Methods). This integration was enabled by extracting the trench-perpendicular component of absolute trench motion estimations from a range of absolute plate motion models [T2019, (*28*, *32*), V2010 (*30*), M2015 (*31*), and D2012 (*33*, *37*)] and considering plate kinematic models including time-dependent plate margin deformation (*26*, *28*, *38*) (Figs. 1B, 2, and 3 and data S1) (see Materials and Methods). The magnitudes of absolute trench motion in both active and ancient flat-slab settings are determined along the flat-slab segments, from their onset age to the present state and from initiation to the time of maximum inland extent, respectively [data S1 (see Materials and Methods)]. Given that, at the onset of flat subduction, the slab hinge aligns with the position of the trench, by estimating the inland extent of the flat slab, the total migration of the slab hinge relative to the trench can be determined (Fig. 1A). From this assessment, it is, therefore, possible to determine the motion of the flat-slab hinge relative to the mantle. Introducing the migration of the slab hinge from the onset of the flat slab as a key component in the analysis allows us to gain a more comprehensive understanding of its dynamics.

**Fig. 5.**
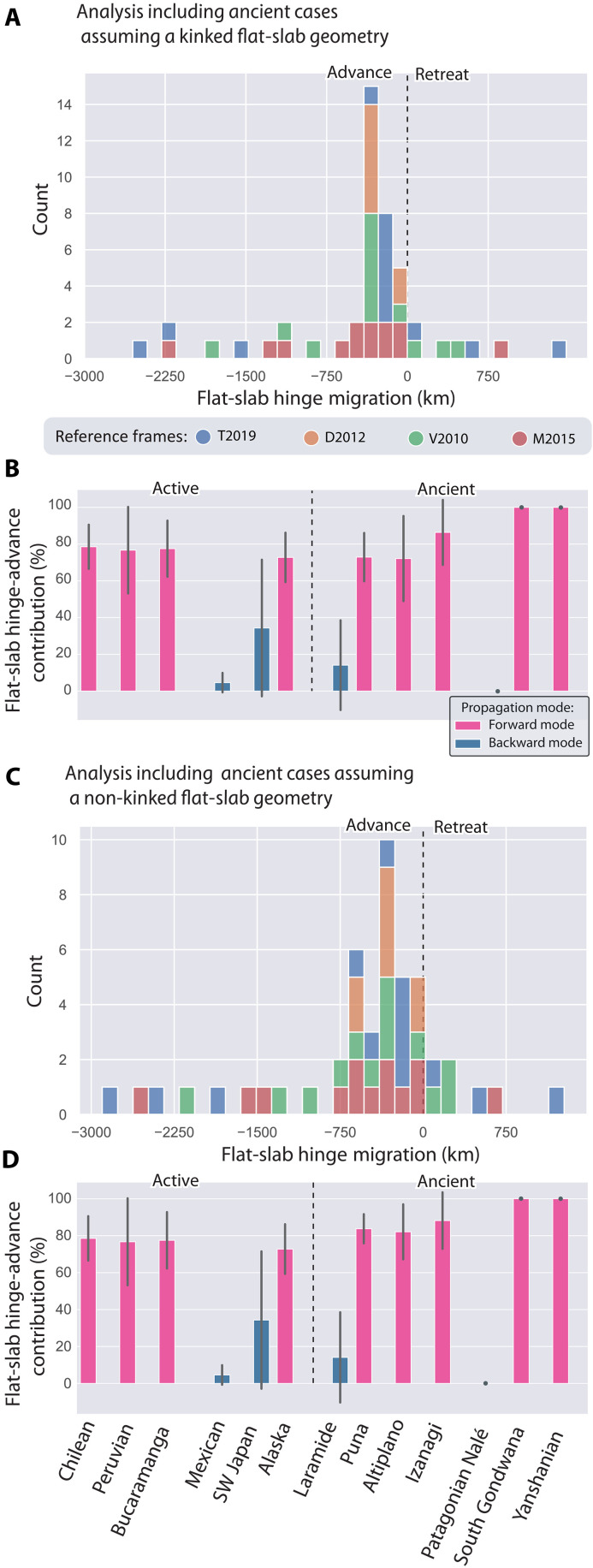
Histogram of absolute flat-slab hinge motion distribution and calculated contributions of flat-slab hinge advance to flat-slab extents. (**A**) Histogram displaying the distribution of absolute flat-slab hinge motions grouped according to the reference frame, showcasing the tendency for hinge-advancing. (**B**) Calculated contributions of absolute flat-slab hinge advance to flat-slab extents. The error bars represent the SD of the calculated values across the different reference frames. Both (A) and (B) consider ancient flat-slab extents determined assuming a kinked flat-slab geometry. The color of the bars denotes the results of *K*-means clustering applied to the percentage contribution of the flat-slab hinge parameter across the different reference frames. (**C** and **D**) Equal to (A) and (B), respectively, but assuming a non-kinked flat-slab geometry in the determination of flat-slab extent for ancient cases (see details in data S1). Reference frames are as follows: T2019 (*28*, *32*); V2010 (*30*); M2015 (*31*); and D2012, a combination of (*37*) and (*33*). SW Japan, Southwest Japan.

Active flat subduction extents are determined directly from current geometries in high-resolution subduction zone models (Fig. 2 and fig. S1) (see Materials and Methods) (*34*). For ancient flat subduction events, the dimension of flat slabs is inferred from time-space arc migration diagrams, which serve as a reliable proxy for potential past flat-slab extents (*3*, *4*, *6*–*9*, *13*–*15*, *24*, *35*, *36*) with arc magmatism closely tracking the leading edge of the progressing flat slab (Figs. 1A and 3 and data S1; see Materials and Methods) (*10*, *39*). This approach has been used since pioneering studies to estimate the potential extents of flat slabs (*3*) and is supported by recent high-resolution numerical models examining magmatic activity evolution and sources in both small-scale (*39*) and large-scale flat subduction (Fig. 3) (*10*). For ease of analysis, we excluded upper-plate shortening in most cases. Compression reduces the distance between the trench and the arc in time-space diagrams, thereby influencing paleo-flat-slab extent calculations. As a result, the estimates presented here represent minimum and, thus, conservative values for our analysis. (Fig. 3). Where data availability permitted, forearc shortening and subduction erosion corrections were incorporated into the calculations (see details in Materials and Methods). The extents of flat slabs in ancient cases are determined using two approaches following the geometries observed in active flat subduction settings: one corresponding to kinked (subhorizontal subduction) geometry and the other to non-kinked (low-angle subduction) geometry, yielding two potential values for each case (denoted as approaches A and B in Fig. 3) (data S1). These values were then incorporated, together with active flat-slab data, into two distinct statistical analyses presented in Figs. 4 and 5. The estimated extents of flat slabs for most of the ancient cases analyzed here are consistent with previous studies, which incorporated additional geological criteria such as the maximum distance of intraplate or broken foreland deformation, dynamic subsidence relocation (*7*, *8*, *10*, *11*, *13*, *14*, *24*, *35*), or the removal of upper-plate mantle lithosphere [e.g., (*9*)]. Consequently, if backward (or forward) propagation dominates the expansion of the flat slab, the statistical analysis should reveal a correlation between trench retreat (or flat-slab hinge motion) and the extent of the flat slabs.

Figures 4 (A to D) and 5 (A to D) present the kinematic framework and the global analysis of the flat-slab database. Statistical analysis of trench motion reveals that, regardless of the reference frame and the assumed approach to measure the potential extents of ancient cases, flat-slab trenches in a flat-slab subduction setting tend to retreat on average, as indicated by positive values (Fig. 4, A and C). The correlation analysis between trench migration and flat-slab extent indicates a negligible relationship, suggesting that, in general, trench retreat does not significantly influence the extent of flat slabs [coefficient of determination (*R*^2^) = 0.00 to 0.0-9, *P* > 0.05; Fig. 4, A and C, and fig. S2]. To further elucidate this, we explored the role of the flat-slab hinge motion in the propagation of the flat slab (Fig. 4, B and D). The flat-slab hinge motion was calculated by subtracting the flat-slab inland extent from the trench motion (negative values imply flat-slab hinge advance) (see details in data S1). The analysis considering a kinked geometry for ancient flat slabs extents indicates that there is a positive relationship between the absolute flat-slab hinge motion and the flat-slab extent, which becomes more evident and significant in certain reference frames [V2010 (*30*) and M2015 (*31*)] (Fig. 4B). This significance is further accentuated and generalized to all reference frames in the analysis considering a non-kinked geometry for ancient flat slabs (Fig. 4D). Notably, this general observation remains significant even when only the highest-resolution active flat-slab settings are considered (*R*^2^ = 0.83 to 0.69, *P* < 0.05; fig. S2). Furthermore, our analyses demonstrate that on average flat-slab hinges tend to advance during the development of flat subduction (Fig. 5, A and C).

We further analyzed the percentage contribution (%) of slab hinge advance (*H*) to flat-slab extent (*F*) across different reference frames, expressed as [H/F]×100 , revealing two distinct behaviors (Fig. 5, B and D). *K*-means clustering was applied to the percentage contribution of flat-slab hinge-advance parameter across the different reference frames (Fig. 5, B and D, and data S1). *K*-means is a clustering algorithm that partitions a set of data points into *K* clusters, where each data point belongs to the cluster with the nearest mean, minimizing the variance within each cluster. This analysis identified two distinct clusters representing extreme end-members. The first cluster, associated with a backward propagation mode, has a mean contribution of 14.1% of hinge advance to flat-slab propagation (SD, 24.8%). These values are consistent across both methods used to measure ancient flat slabs (Fig. 5, B and D). The second cluster, linked to a forward propagation mode, shows mean contributions of 80.6% (83.3%) with SDs of 17% (15%) for the datasets that include kinked (non-kinked) ancient flat-slab extents (Fig. 5, B and D, and data S1).

The predominant clustering of flat slabs associated with dominant forward propagation mode includes the active flat-slab subduction in Colombia, Peru, Chile, and Alaska, as well as the ancient Yanshanian [~185 to 140 million years (Myr)] (*10*, *11*), Izanagi (~100 to 65 Myr) (*9*, *13*, *36*), Altiplano (~50 to 25 Myr), Puna (~30 to 15 Myr) (*7*), and the South Gondwana flat slab (~220 to 195 Myr) (*22*, *35*) (Fig. 5, B and D). Meanwhile, the smaller subset associated with dominant backward propagation includes the active flat subduction zones in Mexico and Southwest Japan, as well as the ancient Western US Laramide flat slab (~90 to 50 Myr) and the Patagonian Nalé flat slab in South America (~120 to 65 Myr) (Fig. 5, B and D). Furthermore, our analysis reveals that most of large-scale flat slabs are ancient, a trend that, with a few exceptions, corresponds to longer times of flat-slab development (fig. S3). Last, space-time diagrams of ancient flat slabs reveal that their development typically occurs in less than 40 Myr (Fig. 3).

## DISCUSSION

### Dominant flat subduction propagation mechanism: Backward or forward mode?

The analysis of the relationships between the inland extent of flat slabs and both absolute trench and flat-slab hinge motion offers key insights into the predominant mechanism driving flat-slab propagation in nature. This study has revealed that the extent of flat slabs can be explained by two end-member models of flat-slab propagation (Figs. 1A, 4, and 5 and fig. S2). However, our results highlight a dominant trend toward the expansion of flat slabs driven by forward propagation (Fig. 5, C and D). This finding holds even when considering only active cases, as evidenced by a statistically significant relationship between flat-slab extent and flat-slab hinge advance (fig. S2). Average contributions of 70 to 75% of forward propagation are observed in four of the six active flat subduction settings (Fig. 5, B and D). These results suggest that, in most of the cases of active and ancient flat slabs analyzed, the lower plate played an active role in propagating flat subduction, contrary to the prevailing notion (Fig. 1A) [e.g., (*16*–*19*, *26*)]. We do not discard a minor contribution of slab angle rotation, limited only to the initiation of flat subduction (*16*, *18*, *24*, *25*).

Independent evidence supporting the forward propagation of flat slabs is observed in Alaska and central Chile. In Alaska, geological and geodetic investigations reveal that the Wrangell block within the upper plate moves in tandem with the underlying flat slab, tracing the motion along the large-scale right-lateral Denali fault [e.g., (*40*)]. In South America, recent regional seismic tomography has revealed a detached slab in the upper mantle beneath the Chilean flat slab, interpreted as a precursor to flat subduction, subsequently overridden by the flat slab (*41*). This unequivocally indicates a forward propagation mode in this active flat slab. Notably, these results also provide further insights into the mechanism of backward propagation. We note that, while the amount of forced trench retreat is similar to the flat-slab length of the Laramide flat slab, effectively meeting the conceptual model by Schepers *et al.* (*26*), in the Patagonian Nalé flat slab, the former largely exceeds the latter (Fig. 4, A and C). This observation implies that, in certain cases, the flat-slab hinge can undergo rollback during the backward propagation of the flat slab, albeit at a slower rate than the imposed trench retreat, thereby facilitating effective flattening of the lower plate (Fig. 1A). Noteworthy, the forward- and backward-propagation modes show a distinct evolution in flat-slab kinematics concerning tectonic and magmatic patterns. In the forward-propagation mode, flat subduction expansion aligns with the migration of the magmatic arc and deformation, as conventionally understood. In contrast, in the backward propagation mode, these trends are opposed (Fig. 1A).

### Driving mechanism behind forward propagating flat subduction

Experimental and conceptual studies suggest that flat-slab formation can result from the upper-plate overthrusting buoyant oceanic lithosphere, often associated with thickened oceanic crust, such as seamount chains or oceanic plateaus (*16*–*19*), or from hydrodynamic suction in the mantle wedge (*25*, *42*), which may be enhanced by cratonic roots or a cold upper plate (*20*, *43*). Our findings suggest a shift in focus toward lower-plate properties and kinematics in explaining flat subduction. Underthrusting, rather than overthrusting, better accounts for most of the analyzed cases (Figs. 4 and 5).

Exploring the formation and longevity of flat slabs goes beyond the scope of this study. However, it is important to highlight this as one facet of the broader issue. As noted by van Hunen *et al.* (*44*), the expansion of flat subduction cannot be fully explained by ridge push force or slab pull acting on the flat-slab hinge. Instead, these forces may be counterbalanced by the trenchward motion of the overriding plate (Fig. 1A). While this mechanism explains backward-propagating flat slabs, the forward propagation modes, dominant along active margins according to our kinematic analysis, require an alternative explanation. Recent studies aiming to elucidate the primary forces driving plate tectonics recognized that both slab pull at subduction zones and convection-induced mantle drag play an important role in the process [e.g., (*45*, *46*)]. Concerning mantle drag, numerical models indicate that mantle flow beneath the subducting plate can induce flat subduction (<10°) (*47*). However, a major limitation of these models is that only small-scale flat slabs are developed, highlighting the need to assess the origin of large-scale flat-slab propagation, as observed in the geological records examined in this study. Alternatively, this external force could be attributed to slab pull from nearby subduction zones [e.g., (*46*)]. Numerical models consistently replicate this external force by imposing variable plate convergence that supplement the spontaneous emergence of slab pull in experiments [e.g., (*25*, *48*)].

**Fig. 6.**
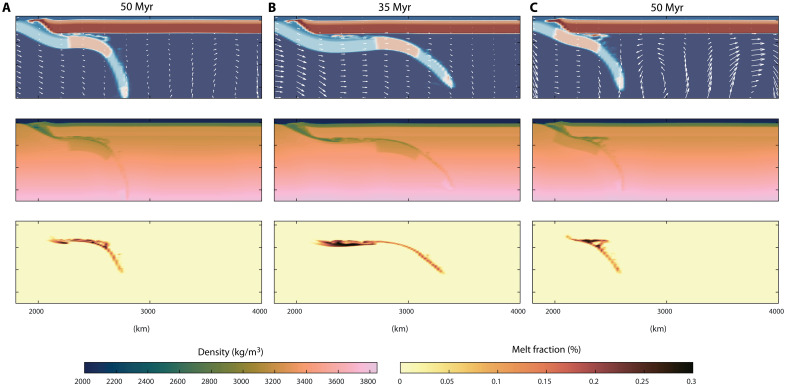
2D geodynamic experiments of flat subduction testing the influence of mantle flow, imposed plate convergence, and slab pull on flat-slab forward propagation. All models begin with an initial phase of imposed convergence (6 cm/year) to initiate subduction and generate a flat-slab geometry over the first 10 Myr. In the second phase, the models diverge: (**A**) Fast mantle flow (5 cm/year) from 200-km depth downward, combined with reduced convergence (2 cm/year), to test the effect of mantle drag under low convergence conditions. (**B**) An average Phanerozoic convergence rate without background mantle flow. (**C**) Both mantle flow and convergence were set to zero, isolating the influence of slab pull driven by negative buoyancy. Parameter choices are described in detail in Materials and Methods and tables S1 and S2. Center and bottom panels show density model distribution and melt fraction computed at the selected time steps for each model.

To assess the relative importance of average ambient mantle flow versus plate convergence in driving the forward propagation of a preexisting flat slab, we conduct a suite of simple 2D thermomechanical experiments in which a buoyant anomaly (e.g., an oceanic plateau or aseismic ridge) has already established a flat subduction configuration (*16*, *18*) (see Materials and Methods and fig. S4 for details in the model setup; see also Fig. 6). Although these models are deliberately simplified and do not capture 3D complexities such as slab curvature or along-strike buoyancy variations, nor do they seek to resolve the ultimate driving mechanisms of flat subduction; they provide a valuable first-order geodynamic framework to investigate forward propagation in flat subduction. Building on and complementing earlier numerical studies [e.g., (*10*, *11*, *16*–*21*, *24*, *25*, *47*)], these experiments allow us to move beyond identifying propagation mechanisms, the primary focus of this study, toward exploring the competing roles of mantle flow and convergence as underlying driving forces, thereby contextualizing our kinematic observations.

Our results indicate that, while mantle flow can drive flat subduction (*47*), it is limited by timescales. Even at fast rates (5 cm/year), the upper bound beneath the Pacific Ocean (*45*), it would take more than 50 Myr to form a large-scale flat slab (Fig. 6A), exceeding the <40-Myr time frame inferred from geological records (Fig. 3). We did not test mantle flow opposing the subduction direction, as it is known to hinder slab shallowing (*43*). The experiment considering a convergence rate of 4 cm/year, consistent with average Phanerozoic plate speeds (*49*) and typically imposed in numerical models as an effect of neighboring slab pull in numerical models [e.g., (*48*)], reproduces observed propagation within realistic timescales (Fig. 6B). Following an initial subduction stage, propagation halts without imposed mantle drag and convergence (Fig. 6C). While not exhaustive, these findings support the hypothesis that imposed plate convergence, likely driven by adjacent slab pull [e.g., (*25*, *48*)], is the primary driver of forward-propagating flat slabs.

In summary, our results demonstrate that flat-slab propagation is governed by distinct end-member processes, with forward propagation, driven primarily by lower-plate underthrusting and bolstered by convergence imposed by the pull of an adjacent slab, emerging as the dominant mode. While upper-plate–driven cases occur, our analyses suggest that they are less common than previously thought.

With substantial advances in geological and geochronological datasets from regions linked to past active margins and the growing recognition of ancient flat slabs, this study offers a timely and robust framework for deciphering flat-slab propagation mechanisms. By introducing a method grounded in geological and kinematic data, it also lays the foundation for more accurate, region-specific models of flat subduction dynamics. Last, as seismologists continue to acquire higher-resolution geophysical data to refine slab geometries or identify additional active flat slabs, this framework will support deeper insights into the processes driving flat subduction.

## MATERIALS AND METHODS

For our analysis, we curated a selection of active and ancient flat-slab cases. In identifying active occurrences, we relied on the pioneering compilation by Gutscher *et al.* (*5*). From this compilation, we specifically selected cases with strong support from recent seismological and geological data sources (*23*, *41*, *50*–*52*). Notably, regions such as New Guinea and Ecuador were excluded because of ongoing debates regarding the subduction angles, whether steep or flat, and the lack of geological constraints on the initiation of flat-slab conditions in these areas, which hampers our kinematic analysis (*34*, *53*). Furthermore, the Costa Rica and Vancouver subduction zones (*5*), if they indeed exhibit flat-slab characteristics, were deemed too incipient and limited in lateral extent (≤200 to 180 km) to warrant inclusion in our analysis. Potential flat subduction in the Caribbean was excluded because of insufficient geophysical constraints on plate geometry and the timing of flat-slab initiation [see discussion by González *et al.* (*54*)]. The Bucaramanga flat slab in northern Colombia (*5*) remains included in our analysis, despite ongoing debates over the interaction between the Nazca and Caribbean subducting plates (*54*). This decision is based on the consensus regarding its existence and formation, dating back to ~9 Myr. This consensus is supported by the spatiotemporal evolution of magmatic arcs, records of upper-plate deformation (*14*, *55*), and recent plate kinematic reconstructions incorporating regional seismic tomography constraints (*54*). Consequently, our analysis focuses on active flat-slab settings in Alaska, Mexico, southwest Japan, Bucaramanga, Peru, and Chile, all of which are well resolved in the Slab2 subduction geometry model and regional geophysical surveys (Fig. 1B and fig. S1) (*23*, *41*, *50*–*52*). Ages of flat-slab onset, based on arc and deformation inland migration (*7*–*11*, *13*, *14*, *22*, *24*, *35*, *56*–*59*), are provided in data S1.

For our analysis of ancient flat subduction cases, we extended our study back only to the Mesozoic era, a period during which plate reconstruction models and mantle reference frames still provide relatively reliable spatial information over time for plate margins (*60*) (see the following subsection for details on our reference frame choices). We incorporated two Cenozoic cases linked to the Altiplano (50 to 25 Myr) and Puna (30 to 18 Myr) flat slabs (*7*, *14*). Additionally, we included five Mesozoic cases encompassing the Laramide (90 to 50 Myr) (*3*, *24*), Yanshanian of East Asia (185 to 140 Myr) (*10*, *11*), Izanagi (100 to 65 Myr) (*9*, *13*, *36*), South Gondwana (220 to 195 Myr) (*22*, *35*), and the Patagonian Nalé flat slab (120 to 65 Myr) (*8*) (see details in table S1) (Figs. 1B and 3). We excluded the Early Triassic flat subduction episode of South China from our analysis (*12*), due to still unreliable constraints on the spatial location of the South China block and its associated plate margin over time (*37*, *60*), which hampers a proper estimation of absolute trench motion.

Flat-slab extents in active settings are obtained directly from the Slab2 subduction zone geometry model (*34*) and a compilation of regional seismic tomography and/or receiver function data available at the flat-slab locations (Fig. 2 and fig. S1). For this analysis, we considered two scenarios on the basis of the observed slab morphology in cross sections from the Slab2 geometry model (*34*) and a compilation of regional seismic tomography and/or receiver function data at flat-slab locations (Fig. 2 and fig. S1) (*23*, *41*, *50*–*52*, *61*). One scenario corresponds to low-angle subduction (i.e., non-kinked flat-slab geometry) and is exemplified by the Alaska and Southwest Japan flat slabs, where the slab maintains a shallow angle of ≤10° or less from the slab hinge near the trench, extending inland for hundreds of kilometers to the sublithospheric slab hinge, where it reenters the mantle (Fig. 2 and fig. S1) (*5*, *40*, *57*, *62*). Hence, for this case, we considered the distance from the trench to the slab hinge, where the maximum flat-slab extent is observed. The other scenario involves subhorizontal subduction (i.e., kinked flat-slab geometry), which occurs in cases with an additional slab kink between the slab hinge near the trench and the deeper slab hinge, where the slab penetrates further into the mantle. In this instance, the extent of the flat slab was determined by measuring the distance from the point where the flat or shallow portion of the slab begins to the distal slab hinge, where the slab reenters the mantle (Fig. 2 and fig. S1). Despite these differences, both geometries are often collectively referred to as flat-slab subduction, as they produce similar tectonic, magmatic, and seismotectonic consequences (*5*) and share a very low angle often less than 10°. The geometric variations arise from the configuration of the upper plate, rather than any specific property of the shallow-dipping lower plate. In the case of the Mexican flat slab, measuring directly from the Slab2 subduction zone geometry model has some limitations, as it only provides information on the remaining flat-slab segment. Previous studies have documented trenchward contraction of this flat-slab hinge after it reached its maximum inland expansion during the Neogene (*56*), suggesting that the flat slab was originally larger than the one observed today.

In ancient flat-slab settings, we used the distance between the reconstructed trench and the furthest inland migration of the magmatic arc, as recorded in space-time diagrams, to evaluate the potential extent of the flat slab before its cessation [e.g., (*3*, *4*)] (Fig. 3 and table S1). Arc positions are influenced by various kinematic and tectonic factors; however, a detailed analysis of the geodynamic processes controlling the time-space evolution of arcs indicates that migrations exceeding 250 km are only explained by changes in subduction geometry (*4*). In this approach, the extents of flat slabs were determined by considering the two possibilities observed in active flat slabs (i.e., kinked and non-kinked flat subduction). For kinked flat subduction, which involves an additional kink near the premigration magmatic arc location, we measured the distance between the arc position before inland migration and the maximum inland location of the magmatic arc (Fig. 3). For non-kinked flat subduction, we directly measured the distance between the reconstructed paleo-trench position and the maximum inland location of the magmatic arc (Fig. 3). In this way, we obtained two potential paleo-flat-slab extents, which were subsequently included, along with active flat-slab data, in two separate statistical analyzes in Figs. 4 and 5. Through this approach, we also determine the time to maximum inland arc migration, which we interpret as the time required for full flat-slab development. This metric is then used to assess absolute trench migration in ancient flat slabs for statistical analyses.

Notably, this approach to determining ancient flat-slab extents is valid primarily for cases where magmatic lulls associated with flat-slab development follow stages of arc migration, although this is not always the case. For instance, the active Peruvian flat slab and its corresponding magmatic arc gap developed without a notable arc migration (*21*). Similarly, for the Late Cretaceous Izanagi flat slab (*13*, *36*), which is included in our analysis, a notable arc gap occurred, and the extent of the flat slab was determined on the basis of the extent of lithospheric mantle removal during flat subduction, which was reproduced in 3D numerical models (*9*).

Therefore, for the specific case of the Izanagi flat-slab episode, we directly use the inferred extent of this flat slab based on previous findings and numerical models presented by Liu *et al.* (*9*) (Fig. 3). We also propose a smaller potential Izanagi flat-slab extent, considering a kinked flat subduction geometry, using a conservative trench-to-arc value of 200 km derived from observations of current settings and the analysis of space-time arc evolution. This value was added to the previous determination of the flat-slab extent to account for the possibility of a shorter flat subduction segment resulting from a kinked flat subduction geometry.

For the specific case of the South Gondwana flat slab (*22*, *35*), when the migration of magmatic arcs ceases, hindering the tracking of the leading edge of the flat slab, we adopt a conservative approach by considering the extent of the flat slab and estimates of trench motion before arc shutoff. Despite this simplification, valuable insights into the underlying mechanisms driving the enlargement of flat slabs are still attainable. It is important to note that the estimated flat-slab extents for most ancient cases analyzed here align with those of previous studies, which used additional geological criteria, such as the maximum distance of intraplate/broken foreland deformation and dynamic subsidence relocation (*7*–*11*, *13*, *14*, *24*, *35*, *63*). We provide the obtained values for the two potential ancient flat-slab extents for each case in data S1.

To construct and analyze time-space magmatic evolution diagrams for ancient flat-slab cases (*3*), we used several previously compiled geochronological datasets (Fig. 3). For the Altiplano, Puna, and South Gondwana flat slabs, we used the Andean geochronological database of igneous rocks compiled by Pilger and hosted by GEOROC (https://data.goettingen-research-online.de/dataset.xhtml?persistentId=doi:10.25625/NGG0Q7). For the Laramide and Yanshanian flat slabs, we used data from the Western US (www.navdat.org) and East Asia, respectively, based on (*10*). In the case of the Patagonian Nalé flat slab, we compiled a new dataset, which is provided in data S2.

Consistent with many previous studies (*3*, *10*, *12*, *24*, *55*), we used this information to construct time-space magmatic evolution diagrams to assess the extent of ancient flat slabs considering the maximum inland extent of magmatic activity. To simplify the analysis, we generally excluded upper-plate shortening, which would, otherwise, reduce the distance between magmatic arc igneous records. As a result, the paleo-flat-slab extent calculations represent minimum estimates only (Fig. 3). As in most cases, we did not consider forearc shortening and potential subduction erosion in our space-time diagrams; hence, the paleo-flat-slab extent calculations represent only minimum values (Fig. 3). The Cenozoic Andean flat slabs are exceptions, where subduction erosion and forearc shortening are important and relatively well quantified (*64*, *65*). To account for these processes, we first calculated the time since the demise of flat slab to the present day, along with average subduction erosion rates of 1.5 km/Myr for the study area (*64*). We then incorporated a maximum forearc shortening of 44 km, recently estimated by Habel *et al.* (*65*), resulting in net corrections of arc-trench distances of 81.5 and 71 km for the Altiplano and Puna flat slabs, respectively.

### Plate kinematic reconstructions and selection of reference frames

To better understand how flat slabs enlarge within a kinematic framework, we integrated our global database with the dynamics of the underlying mantle. This approach allows us to establish relationships between the length of the flat slab, trench migration, and the motion of the flat-slab hinge. We used PyGPlates, an open-source Python library, to interact with plate tectonic reconstruction models (www.gplates.org).

Describing the movement of tectonic plates over geological time requires considering both their relative and absolute motions. While relative plate motions have been resolved with high precision over the past few decades [e.g., (*29*)], their description relative to the underlying mantle depends on the absolute plate motion model. This variation arises because different methodologies and absolute reference frames have been used to describe plate and plate boundary motion [e.g., (*30*–*33*, *66*, *67*)].

To quantify the normal component of absolute trench migration and the motion of the flat-slab hinge, we explored several absolute plate motion models and assessed the uncertainty in our observations. We used the relative plate motion model of Seton *et al.* (*29*) as the baseline. For critical regions, such as the South American plate margin, which has undergone important modifications and hosts most of our analyzed flat-slab occurrences, we incorporated the evolving plate margin model from Schepers *et al.* (*26*).

For the Bucaramanga flat slab in northern South America, we considered the high-resolution plate margin deformation model developed by Montes *et al.* (*38*). In the case of the Alaska flat slab, we observed a difference in trench location on the North American plate between its current position and that shown in the models by Seton *et al.* (*29*) and Müller *et al.* (*28*). Although this disparity does not affect the absolute magnitude of trench motion, it does affect the reconstructed position of the trench at the onset of flat-slab subduction. Also, we included the plate kinematic model by Müller *et al.* (*28*), which provides time-dependent plate margin deformation for several active margins.

In our evaluation of absolute plate motion models, we considered mantle reference frames, which are based on different approaches and assumptions and provide information on paleo-latitudes and longitudes (Figs. 4 and 5). Traditional methods for defining absolute plate motion rely on hot-spot tracks that display clear age progressions, representing plate motions over mantle plumes ascending from the deep mantle. Hot spots used to constrain absolute plate motion models can be considered either fixed or mobile [e.g., (*68*)]. Maher *et al.* (*31*) (hereafter, M2015) established an absolute plate motion model relative to Africa by using hot-spot tracks within the Indian and Atlantic Oceans. This methodology assumed the relative stability of hot spots within the Indo-Atlantic region from 82 Myr to the present day. This assumption is considered suitable for the study of active and ancient flat slabs in North, Central, and South America, based on the finding by O’Neill *et al.* (*67*) that predictions from both moving and fixed hot-spot reference frames are not significantly different within their uncertainties since 80 Myr. Efforts to reconcile absolute plate motions with hot-spot movement have led to the development of global moving hot-spot models (*68*). These models aim to reconcile the age-progressive volcanic trails across the Indian, Atlantic, and Pacific Ocean basins. Hence, we included the global moving hot-spot reference frame proposed by Doubrovine *et al.* (*33*) after 120 Myr, and, before that, a paleomagnetic reference frame adjusted for True Polar Wander by Steinberger and Torsvik (*66*), integrated into the Paleozoic plate kinematic model of Matthews *et al.* (*37*). The rationale for this choice is to obtain absolute trench motion estimates from at least three different reference frames for the Triassic-Jurassic South Gondwana and Yanshanian flat-slab events. For practicality, we present these results jointly as D2012 in Figs. 4 and 5 and data S1.

We also used the slab-fitted reference frame introduced by van der Meer *et al.* (*30*), which connects surface plate motions to subducted slab remnants. This frame is built upon the paleomagnetic framework provided by Torsvik *et al.* (*69*) and incorporates constraints on paleolongitude derived from subducted slab remnants mapped using seismic tomography. Additionally, we used the reference frame proposed by Tetley *et al.* (*32*), hereafter referred to as T2019, which is integrated into the plate kinematic model developed by Müller *et al.* (*28*). This reference frame incorporates age-progressive hot-spot tracks, subduction zone migration, and rates of net lithospheric rotation within an optimization framework called tectonic rules–based plate-motion model optimization. It is important to note that the extent of analysis varied across cases because of the limitations inherent in certain reference frames. For instance, hot spot–related reference frames cannot extend beyond the Late Early Cretaceous [up to 83 Myr for fixed hot spots (*31*) and 120 Myr for moving hot spots (*33*)]. As a result, for some absolute reference frames, certain flat slabs may lack absolute trench motion data.

For active flat slabs, trench-perpendicular absolute trench motion magnitudes were assessed from the onset of flat-slab development, as reported in the literature (see data S1), to the present day. A specific case is the active Mexican flat slab, whose flat-slab hinge has been retreating since 7 Myr due to flat-slab destabilization (*56*). Therefore, for this case, trench motion was evaluated from the onset of development at 20 to 7 Myr (*56*). For ancient flat slabs, this parameter was evaluated from the time of initial arc migration to the point of maximum inland arc location, representing the full development of the flat slab (Fig. 2) (*4*). We present the calculated values for absolute trench motion and flat-slab hinge motion as averages derived from measurements at three locations: the center and both extremities of the ancient and active flat slabs. These values are provided in data S1. Last, our results, indicating a major component of forward propagation in the Peru and Chile flat slabs, differ from the previous conclusions by Schepers *et al.* (*26*). This discrepancy arises from the incorporation of the updated global subduction model Slab2 (*34*), supported by higher-resolution regional subduction geometry models (see fig. S1). Our study integrates regional seismic tomography to refine the locations of active flat slabs and uses a broader analysis of absolute trench motion, using multiple absolute plate motion models.

### Numerical modeling of flat subduction

To evaluate the potential driving forces behind the forward propagation of flat subduction, we develop 2D thermomecanichal subduction models. These geodynamic models are specifically designed to investigate mantle flow and plate convergence (as imposed by neighboring active subduction [e.g., (*48*)] as the two potential drivers of forward propagation of flat subduction while balancing computational feasibility and physical realism. The equations governing the conservation of mass, momentum, and energy are solved for an incompressible, viscoplastic fluid within a 2D Cartesian domain. We use the finite element, particle-in-cell code Underworld2 (*70*–*72*) to carry out these computations. This code uses a continuum mechanics approximation, a widely applied method to describe geological and geophysical processes and to solve the conservation equations of momentum, mass, and energy

$$\nabla \cdot \left(\eta \nabla^{s}u\right)+\nabla p=\rho g$$ (1)

∇⋅u=0 (2)

$$\rho C_{p}\left(\frac{\partial T}{\partial t}+u\nabla T\right)=\nabla \cdot \left(k\nabla T\right)+\rho f$$ (3)

where u is the velocity, the $\nabla^{s}=\frac{1}{2}\left(\nabla +\nabla^{T}\right)$ is the symmetrized gradient operator, *T* the temperature, *p* the pressure, η is the dynamic viscosity, ρ is the density, g is the gravitational acceleration vector, *C_p_* is the isobaric heat capacity, *k* is the thermal conductivity, and *f* is a heat source term accounting for the radiogenic heating, adiabatic heating and shear heating.

We use a viscoplastic rheology that depends on nonlinear temperature and strain rate. The viscous deformation of rocks is computed using a power-law equation, where dislocation and diffusion creep are characterized by a generic stress-strain rate relationship for each mechanism

$$\dot{\varepsilon }=A{\left(\sigma^{\prime }/\mu \right)}^{n}{\left(b/d\right)}^{m}\text{exp}\left(-\frac{E+\mathrm{PV}}{\mathrm{RT}}\right)$$ (4)

where *d* is the average grain-size, σ is the deviatoric stress second invariant, *A* is the pre-exponential factor, μ is the shear modulus, *b* is the length of the Burgers vector (i.e., a measure of lattice distortion), *n* is the stress exponent, *m* is the grain-size exponent, *E* is the activation energy, *V* is the activation volume, and *R* is the gas constant. Viscosity in the model is constrained between 10^19^ and 10^24^ Pa·s. Maximum strain rates in the model reach ~10^−14^ s^−1^, yielding viscosities greater than 10^19^ Pa·s. Table S1 describes the dimensional values used in this study.

We have implemented a melt function to account for the thermal and mechanical effects of partial melting (*M*). This is consistent with magmatic activity in this specific geodynamic setting and simulates the relative decoupling between the plates that facilitates the lateral growth of flat subduction. However, this function does not account for melt extraction processes. Thus, the code is restricted to partially melted regions in which the melt remains in situ. The mechanical effect of partial melting is represented by a viscosity reduction in the lower crust within a melt range of 0.15 to 0.3. Melting modifies the existing viscous rheology, calculated as

$$M_{\text{int}}=1+\left(\frac{M_{f}-L_{f}}{L_{f}-U_{f}}\right)$$ (5)

$$\eta_{m}=\eta \times \left[1+M_{\text{int}}+\eta_{f}\times \left(1-M_{\text{int}}\right)\right]$$ (6)

where η*_m_* is the effective viscosity after melting, η is the viscous rheology, and *M*_int_ denotes a normalized linear interpolation of the percentage of the melt fraction (*M*_f_) between the upper limit (*U*_f_ = 30%) and lower limit (*L*_f_ = 15%) of the melt fraction range, and η_f_ is the melt viscous softening factor. As the melt fraction increases from 15 to 30%, the viscosity decreases by two orders of magnitude (*73*). The melt fraction (*M*_f_) is a function of temperature and is calculated as

$$T_{\text{ss}}=\frac{\left[T-\left(T_{s}+T_{l}\right)\times 0.5\right]}{\left(T_{l}-T_{s}\right)}$$ (7)

$$\begin{array}{c} M_{f}=0.5+T_{\text{ss}}+\left(T_{\text{ss}}^{2}-0.25\right)\times \left(0.4256+2.988\times T_{\text{ss}}\right) \end{array}$$ (8)

Here, *T*_ss_ represents the super-solidus temperature, *T*_s_ denotes the solidus temperature, and *T*_l_ is the liquidus temperature. Both *T*_s_ and *T*_l_ are temperature and pressure dependent and defined by quadratic polynomials

$$T_{s}=a_{s}+b_{s}P+c_{s}P^{2}$$ (9)

$$T_{l}=a_{l}+b_{l}P+c_{l}P^{2}$$ (10)

where *a*, *b*, and *c* are constants and are defined in table S2.

Plastic failure is governed by a pressure-dependent Drucker-Prager yield criterion. The brittle properties of materials evolve with strain, such that both cohesion and the friction coefficient decrease linearly with accumulated plastic strain. In our simulations, the yield stress decreases linearly to a maximum of 20% of its initial value (or to 2 MPa) when the accumulated strain reaches 0.5 for all materials.

The initial model setup geometry is illustrated in fig. S4, and the material properties and model parameters are specified in table S2. The model domain is 2D, measuring 6000 km in width and 660 km in depth. The 2D assumption is used as a first-order approximation of the complex geodynamic processes under investigation. While a fully 3D spherical model would provide additional insights, the computational cost is higher, and previous studies [e.g., (*10*, *11*, *16*, *17*, *20*, *21*, *25*)] have shown that 2D models can effectively capture the primary physics of flat subduction.

The model uses a free-slip condition on the top boundary (*u_y_* = 0) and free-outflow conditions at the bottom. Our models focus on upper-mantle and lithospheric processes; hence, the lower mantle is excluded to simplify computations and to focus on the primary mechanisms driving flat-slab subduction. A uniform grid is used, consisting of 256 × 128 nodal points. Initially, the configuration represents an oceanic lithosphere with a 45° dipping weak zone within the mantle lithosphere at *x* = 2000 km where subduction is initiated and including a relatively buoyant domain of 3100 kg m^−3^ of 500 × 90 km simulating an oceanic plateau (fig. S4). The inclusion of this feature intended to facilitate flat subduction [e.g., (*16*, *17*, *25*)], which is then propagated by either mantle flow or imposed convergence in our experiments (see details in the following paragraph). This approach aligns with our primary objective of assessing the relative influence of these two factors on the forward propagation of flat slabs. Following the approach of Beaumont *et al.* (*74*), we adopt a set of laboratory-derived rheological parameters. The continental upper crust exhibit a wet quartzite rheology (*75*), while the continental lower crust follows a dry Maryland diabase rheology (*76*). The oceanic crust and oceanic mantle lithosphere are characterized by the rheology of wet olivine (*77*). The weak zone also has a wet olivine rheology with a low friction coefficient, while the continental mantle lithosphere adopts a dry olivine rheology. Water content is introduced for the oceanic crust and weak zone rheology to facilitate the onset of subduction. Additionally, a 30-km “sticky air” layer with low viscosity (10^19^ Pa s) and density (1 kg m^−3^) is included to minimize shear stresses at the surface and create a pseudo free surface. A constant temperature (*T* = 0°C) is imposed at the top boundary with no heat flux across the side walls. Initially, the internal temperature distribution follows a geothermal gradient of 13°C km^−1^ until reaching a temperature of 1300°C at the lithosphere-asthenosphere boundary (LAB) at a depth of 100 km (fig. S4). Below the base of the LAB, temperatures are interpolated linearly between 1300° and 1573°C.

Our modeling approach consists of three main experiments, each beginning with an initial phase that imposes a convergence rate of 6 cm/year to initiate subduction and establish a flat-slab geometry. This setup allows us to test the roles of mantle flow, convergence, and slab pull in flat-slab forward propagation (Fig. 6). During this first phase, convergence is applied across the lithosphere from the left boundary for the first 10 Myr. In the second phase, the models diverge. In model A (Fig. 6A), we assess the effect of fast mantle flow (5 cm/year), consistent with Pacific Ocean estimates (*45*), combined with reduced convergence (2 cm/year). Mantle flow is applied from 200-km depth to the model base, enabling mantle drag under low convergence conditions. An outflow matching the imposed inflow is set at the lower right corner to minimize boundary effects and ensure a physically consistent velocity field. This configuration represents background mantle flow (*47*), with the outflow placed far from the region of interest to avoid artificial interference. Because all flat slabs analyzed, both active and ancient, are or were located within the Pacific Ring of Fire, testing flow rates beyond existing estimates (*45*) would be inconsistent with geodynamic constraints and of limited value for comparison. In model B (Fig. 6B), we impose an average Phanerozoic plate convergence rate without mantle flow. Our use of average convergence values derived from global plate tectonic speed-limit analyses (*49*) already reproduces realistic propagation rates, making further parameter testing unnecessary (Fig. 6C). In model C (Fig. 6C), both mantle flow and convergence are set to zero to isolate the effect of slab pull driven by negative buoyancy.

## Supplementary Materials

### The PDF file includes:

Figs. S1 to S4 Tables S1 and S2 Legends for movies S1 to S3 Legends for data S1 and S2 References

## References

[1] M. Barazangi, B. L. Isacks, Spatial distribution of earthquakes and subduction of the Nazca plate beneath South America. Geology 4, 686–692 (1976).

[2] B. L. Isacks, M. Barazangi, “Geometry of Benioff zones: Lateral segmentation and downwards bending of the subducted lithosphere,” in *Island Arcs, Deep Sea Trenches and Back-Arc Basins* (John Wiley & Sons, 1977), vol. 1, pp. 99–114.

[3] P. J. Coney, S. J. Reynolds, Cordilleran benioff zones. Nature 270, 403–406 (1977).

[4] G. M. Gianni, S. P. Luján, Geodynamic controls on magmatic arc migration and quiescence. Earth Sci. Rev. 218, 103676 (2021).

[5] M.-A. Gutscher, W. Spakman, H. Bijwaard, E. R. Engdahl, Geodynamics of flat subduction: Seismicity and tomographic constraints from the Andean margin. Tectonics 19, 814–833 (2000).

[6] V. A. Ramos, E. O. Cristallini, D. J. Pérez, The pampean flat-slab of the central andes. J. South Am. Earth Sci. 15, 59–78 (2002).

[7] J. Martinod, M. Gérault, L. Husson, V. Regard, Widening of the Andes: An interplay between subduction dynamics and crustal wedge tectonics. Earth Sci. Rev. 204, 103170 (2020).

[8] G. M. Gianni, F. M. Dávila, A. Echaurren, L. Fennell, J. Tobal, C. Navarrete, P. Quezada, A. Folguera, M. Giménez, A geodynamic model linking Cretaceous orogeny, arc migration, foreland dynamic subsidence and marine ingression in southern South America. Earth Sci. Rev. 185, 437–462 (2018).

[9] L. Liu, D. Peng, L. Liu, L. Chen, S. Li, Y. Wang, Z. Cao, M. Feng, East Asian lithospheric evolution dictated by multistage Mesozoic flat-slab subduction. Earth Sci. Rev. 217, 103621 (2021).

[10] L. Liu, L. Liu, Y.-G. Xu, Mesozoic intraplate tectonism of East Asia due to flat subduction of a composite terrane slab. Earth Sci. Rev. 214, 103505 (2021).

[11] F.-Y. Wu, J.-H. Yang, Y.-G. Xu, S. A. Wilde, R. J. Walker, Destruction of the North China craton in the Mesozoic. Annu. Rev. Earth Planet. Sci. 47, 173–195 (2019).

[12] Z.-X. Li, X.-H. Li, Formation of the 1300-km-wide intracontinental orogen and postorogenic magmatic province in Mesozoic South China: A flat-slab subduction model. Geology 35, 179–182 (2007).

[13] D. Peng, L. Liu, Y. Wang, A newly discovered Late-Cretaceous East Asian flat slab explains its unique lithospheric structure and tectonics. J. Geophys. Res. Solid Earth 126, e2021JB022103 (2021).

[14] V. A. Ramos, A. Folguera, Andean flat-slab subduction through time. Geol. Soc. Lond. Spec. Publ. 327, 31–54 (2009).

[15] A. Folguera, V. A. Ramos, Repeated eastward shifts of arc magmatism in the Southern Andes: A revision to the long-term pattern of Andean uplift and magmatism. J. South Am. Earth Sci. 32, 531–546 (2011).

[16] J. van Hunen, A. P. van den Berg, N. J. Vlaar, The impact of the South-American plate motion and the Nazca Ridge subduction on the flat subduction below South Peru. Geophys. Res. Lett. 29, 35-1–35-4 (2002).

[17] J. van Hunen, A. P. van den Berg, N. J. Vlaar, Various mechanisms to induce present-day shallow flat subduction and implications for the younger Earth: A numerical parameter study. Phys. Earth Planet. In. 146, 179–194 (2004).

[18] N. Espurt, F. Funiciello, J. Martinod, B. Guillaume, V. Regard, C. Faccenna, S. Brusset, Flat subduction dynamics and deformation of the South American plate: Insights from analog modeling. Tectonics 27, TC3011 (2008).

[19] J. Martinod, B. Guillaume, N. Espurt, C. Faccenna, F. Funiciello, V. Regard, Effect of aseismic ridge subduction on slab geometry and overriding plate deformation: Insights from analogue modeling. Tectonophysics 588, 39–55 (2013).

[20] V. C. Manea, M. Pérez-Gussinyé, M. Manea, Chilean flat slab subduction controlled by overriding plate thickness and trench rollback. Geology 40, 35–38 (2012).

[21] J. Hu, L. Liu, A. Hermosillo, Q. Zhou, Simulation of late Cenozoic South American flat-slab subduction using geodynamic models with data assimilation. Earth Planet. Sci. Lett. 438, 1–13 (2016).

[22] G. M. Gianni, C. Navarrete, S. Spagnotto, Surface and mantle records reveal an ancient slab tear beneath Gondwana. Sci. Rep. 9, 19774 (2019). 31875052 10.1038/s41598-019-56335-9 PMC6930287

[23] B. T. Bishop, S. L. Beck, G. Zandt, L. S. Wagner, M. D. Long, H. Tavera, Foreland uplift during flat subduction: Insights from the Peruvian Andes and Fitzcarrald Arch. Tectonophysics 731-732, 73–84 (2018).

[24] P. Copeland, C. A. Currie, T. F. Lawton, M. A. Murphy, Location, location, location: The variable lifespan of the Laramide orogeny. Geology 45, 223–226 (2017).

[25] S. Liu, C. A. Currie, Farallon plate dynamics prior to the Laramide orogeny: Numerical models of flat subduction. Tectonophysics 666, 33–47 (2016).

[26] G. Schepers, D. J. J. van Hinsbergen, W. Spakman, M. E. Kosters, L. M. Boschman, N. McQuarrie, South-American plate advance and forced Andean trench retreat as drivers for transient flat subduction episodes. Nat. Commun. 8, 15249 (2017). 28508893 10.1038/ncomms15249 PMC5440808

[27] M. Barazangi, B. L. Isacks, Subduction of the Nazca plate beneath Peru: Evidence from spatial distribution of earthquakes. Geophys. J. Int. 57, 537–555 (1979).

[28] R. D. Müller, S. Zahirovic, S. E. Williams, J. Cannon, M. Seton, D. J. Bower, M. G. Tetley, C. Heine, E. le Breton, S. Liu, S. H. J. Russell, T. Yang, J. Leonard, M. Gurnis, A global plate model including lithospheric deformation along major rifts and orogens since the Triassic. Tectonics 38, 1884–1907 (2019).

[29] M. Seton, R. D. Müller, S. Zahirovic, C. Gaina, T. Torsvik, G. Shephard, A. Talsma, M. Gurnis, M. Turner, S. Maus, M. Chandler, Global continental and ocean basin reconstructions since 200 Ma. Earth Sci. Rev. 113, 212–270 (2012).

[30] D. G. van Der Meer, W. Spakman, D. J. Van Hinsbergen, M. L. Amaru, T. H. Torsvik, Towards absolute plate motions constrained by lower-mantle slab remnants. Nat. Geosci. 3, 36–40 (2010).

[31] S. Maher, P. Wessel, R. Müller, S. Williams, Y. Harada, Absolute plate motion of Africa around Hawaii-Emperor bend time. Geophys. J. Int. 201, 1743–1764 (2015).

[32] M. G. Tetley, S. E. Williams, M. Gurnis, N. Flament, R. D. Müller, Constraining absolute plate motions since the Triassic. J. Geophys. Res. Solid Earth 124, 7231–7258 (2019).

[33] P. V. Doubrovine, B. Steinberger, T. H. Torsvik, Absolute plate motions in a reference frame defined by moving hot spots in the Pacific, Atlantic, and Indian oceans. J. Geophys. Res. Solid Earth 117, B09101 (2012).

[34] G. P. Hayes, G. L. Moore, D. E. Portner, M. Hearne, H. Flamme, M. Furtney, G. M. Smoczyk, Slab2, a comprehensive subduction zone geometry model. Science 362, 58–61 (2018). 30093602 10.1126/science.aat4723

[35] C. Navarrete, G. Gianni, A. Encinas, M. Márquez, Y. Kamerbeek, M. Valle, A. Folguera, Triassic to Middle Jurassic geodynamic evolution of southwestern Gondwana: From a large flat-slab to mantle plume suction in a rollback subduction setting. Earth Sci. Rev. 194, 125–159 (2019).

[36] Y. Liu, L. Liu, Z. Wu, W. Li, X. Hao, New insight into East Asian tectonism since the late Mesozoic inferred from erratic inversions of NW-trending faulting within the Bohai Bay Basin. Gondw. Res. 102, 17–30 (2022).

[37] K. J. Matthews, K. T. Maloney, S. Zahirovic, S. E. Williams, M. Seton, R. D. Müller, Global plate boundary evolution and kinematics since the late Paleozoic. Global Planet. Change 146, 226–250 (2016).

[38] C. Montes, A. F. Rodriguez-Corcho, G. Bayona, N. Hoyos, S. Zapata, A. Cardona, Continental margin response to multiple arc-continent collisions: The northern Andes-Caribbean margin. Earth Sci. Rev. 198, 102903 (2019).

[39] X. Liu, L. S. Wagner, C. A. Currie, M. J. Caddick, Implications of flat-slab subduction on hydration, slab seismicity, and arc volcanism in the Pampean region of Chile and Argentina. Geochem. Geophys. Geosyst. 25, e2023GC011317 (2024).

[40] M. A. Jadamec, M. I. Billen, S. M. Roeske, Three-dimensional numerical models of flat slab subduction and the Denali fault driving deformation in south-central Alaska. Earth Planet. Sci. Lett. 376, 29–42 (2013).

[41] Y. Gao, X. Yuan, B. Heit, F. Tilmann, D. P. van Herwaarden, S. Thrastarson, A. Fichtner, B. Schurr, Impact of the Juan Fernandez Ridge on the Pampean flat subduction inferred from full waveform inversion. Geophys. Res. Lett. 48, e2021GL095509 (2021).

[42] D. Stevenson, J. Turner, Angle of subduction. Nature 270, 334–336 (1977).

[43] J. Rodríguez-González, A. M. Negredo, M. I. Billen, The role of the overriding plate thermal state on slab dip variability and on the occurrence of flat subduction. Geochem. Geophys. Geosyst. 13, Q01002 (2012).

[44] J. van Hunen, A. P. van den Berg, N. J. Vlaar, A thermo-mechanical model of horizontal subduction below an overriding plate. Earth Planet. Sci. Lett. 182, 157–169 (2000).

[45] D. B. Rowley, A. M. Forte, C. J. Rowan, P. Glišović, R. Moucha, S. P. Grand, N. A. Simmons, Kinematics and dynamics of the East Pacific Rise linked to a stable, deep-mantle upwelling. Sci. Adv. 2, e1601107 (2016). 28028535 10.1126/sciadv.1601107 PMC5182052

[46] D. Forsyth, S. Uyeda, On the relative importance of the driving forces of plate motion. Geophys. J. Int. 43, 163–200 (1975).

[47] J. Rodríguez-González, A. M. Negredo, E. Carminati, Slab-mantle flow interaction: Influence on subduction dynamics and duration. Terra Nova 26, 265–272 (2014).

[48] P. Huangfu, Y. Wang, P. A. Cawood, Z. H. Li, W. Fan, T. V. Gerya, Thermo-mechanical controls of flat subduction: Insights from numerical modeling. Gondw. Res. 40, 170–183 (2016).

[49] S. Zahirovic, R. D. Müller, M. Seton, N. Flament, Tectonic speed limits from plate kinematic reconstructions. Earth Planet. Sci. Lett. 418, 40–52 (2015).

[50] R. Martin-Short, R. Allen, I. D. Bastow, R. W. Porritt, M. S. Miller, Seismic imaging of the Alaska subduction zone: Implications for slab geometry and volcanism. Geochem. Geophys. Geosyst. 19, 4541–4560 (2018).

[51] Y. Kim, R. W. Clayton, J. M. Jackson, Geometry and seismic properties of the subducting Cocos plate in central Mexico. J. Geophys. Res. 115, B06310 (2010).

[52] C. Chiarabba, P. de Gori, C. Faccenna, F. Speranza, D. Seccia, V. Dionicio, G. A. Prieto, Subduction system and flat slab beneath the Eastern Cordillera of Colombia. Geochem. Geophys. Geosyst. 17, 16–27 (2016).

[53] D. J. van Hinsbergen, W. Spakman, H. de Boorder, M. van Dongen, S. M. Jowitt, P. R. D. Mason, Arc-type magmatism due to continental-edge plowing through ancient subduction-enriched mantle. Geophys. Res. Lett. 47, e2020GL087484 (2020).

[54] R. González, O. Oncken, C. Faccenna, E. le Breton, M. Bezada, A. Mora, Kinematics and convergent tectonics of the Northwestern South American plate during the Cenozoic. Geochem. Geophys. Geosyst. 24, e2022GC010827 (2023).

[55] L. Wagner, J. S. Jaramillo, L. F. Ramírez-Hoyos, G. Monsalve, A. Cardona, T. W. Becker, Transient slab flattening beneath Colombia. Geophys. Res. Lett. 44, 6616–6623 (2017).

[56] V. C. Manea, M. Manea, L. Ferrari, T. Orozco-Esquivel, R. W. Valenzuela, A. Husker, V. Kostoglodov, A review of the geodynamic evolution of flat slab subduction in Mexico, Peru, and Chile. Tectonophysics 695, 27–52 (2017).

[57] E. J. Moreno, V. C. Manea, M. Manea, S. Yoshioka, N. Suenaga, A. Bayona, Numerical modeling of subduction and evaluation of Philippine Sea Plate tectonic history along the Nankai Trough. Sci. Rep. 13, 18313 (2023). 37880308 10.1038/s41598-023-45370-2 PMC10600142

[58] J. M. Trop, J. A. Benowitz, C. S. Kirby, M. E. Brueseke, Geochronology of the Wrangell Arc: Spatial-temporal evolution of slab-edge magmatism along a flat-slab, subduction-transform transition, Alaska-Yukon. Geosphere 18, 19–48 (2022).

[59] T. Waldien, R. O. Lease, S. Roeske, J. Benowitz, P. O’Sullivan, The role of preexisting upper plate strike-slip faults during long-lived (ca. 30 Myr) oblique flat slab subduction, southern Alaska. Earth Planet. Sci. Lett. 577, 117242 (2022).

[60] T. H. Torsvik, L. R. M. Cocks, The integration of palaeomagnetism, the geological record and mantle tomography in the location of ancient continents. Geol. Mag. 156, 242–260 (2019).

[61] K. Asamori, D. Zhao, Teleseismic shear wave tomography of the Japan subduction zone. Geophys. J. Int. 203, 1752–1772 (2015).

[62] E. S. Finzel, J. M. Trop, K. D. Ridgway, E. Enkelmann, Upper plate proxies for flat-slab subduction processes in southern Alaska. Earth Planet. Sci. Lett. 303, 348–360 (2011).

[63] L. Liu, M. Gurnis, M. Seton, J. Saleeby, R. D. Müller, J. M. Jackson, The role of oceanic plateau subduction in the Laramide orogeny. Nat. Geosci. 3, 353–357 (2010).

[64] E. Scheuber, K.-J. Reutter, Magmatic arc tectonics in the Central Andes between 21 and 25 S. Tectonophysics 205, 127–140 (1992).

[65] T. Habel, M. Simoes, R. Lacassin, D. Carrizo, G. Aguilar, A contribution to the quantification of crustal shortening and kinematics of deformation across the Western Andes (∼ 20–22° S). Solid Earth 14, 17–42 (2023).

[66] B. Steinberger, T. H. Torsvik, Absolute plate motions and true polar wander in the absence of hotspot tracks. Nature 452, 620–623 (2008). 18385737 10.1038/nature06824

[67] C. O’Neill, D. Müller, B. Steinberger, On the uncertainties in hot spot reconstructions and the significance of moving hot spot reference frames. Geochem. Geophys. Geosyst. 6, Q04003 (2005).

[68] B. Steinberger, R. Sutherland, R. J. O’connell, Prediction of Emperor-Hawaii seamount locations from a revised model of global plate motion and mantle flow. Nature 430, 167–173 (2004). 15241405 10.1038/nature02660

[69] T. H. Torsvik, R. D. Müller, R. Van der Voo, B. Steinberger, C. Gaina, Global plate motion frames: Toward a unified model. Rev. Geophys. 46, RG3004 (2008).

[70] R. Beucher, L. Moresi, J. Giordani, J. Mansour, D. Sandiford, R. Farrington, L. Mondy, C. Mallard, P. Rey, G. Duclaux, O. Kaluza, A. Laik, S. Morón, UWGeodynamics: A teaching and research tool for numerical geodynamic modelling. J. Open Source Softw. 4, 1136 (2019).

[71] L. Moresi, F. Dufour, H.-B. Mühlhaus, A Lagrangian integration point finite element method for large deformation modeling of viscoelastic geomaterials. J. Comput. Phys. 184, 476–497 (2003).

[72] L. Moresi, S. Quenette, V. Lemiale, C. Mériaux, B. Appelbe, H. B. Mühlhaus, Computational approaches to studying non-linear dynamics of the crust and mantle. Phys. Earth Planet. Inter. 163, 69–82 (2007).

[73] C. Rosenberg, M. Handy, Experimental deformation of partially melted granite revisited: Implications for the continental crust. J. Metam. Geol. 23, 19–28 (2005).

[74] C. Beaumont, M. Nguyen, R. A. Jamieson, S. Ellis, Crustal flow modes in large hot orogens. Geol. Soc. Spec. Publ. 268, 91–145 (2006).

[75] G. C. Gleason, J. Tullis, A flow law for dislocation creep of quartz aggregates determined with the molten salt cell. Tectonophysics 247, 1–23 (1995).

[76] S. Mackwell, M. Zimmerman, D. Kohlstedt, High-temperature deformation of dry diabase with application to tectonics on Venus. J. Geophys. Res. Solid Earth 103, 975–984 (1998).

[77] S.-I. Karato, P. Wu, Rheology of the upper mantle: A synthesis. Science 260, 771–778 (1993). 17746109 10.1126/science.260.5109.771

[78] G. M. Gianni, L. Gallo, Jupyter Notebooks and files to accompany the paper: Slab underthrusting is the primary control on flat slab size, Zenodo (2025); 10.5281/zenodo.15558004. PMC12248310 40644538

[79] A. C. Adriasola, S. N. Thomson, M. R. Brix, F. Hervé, B. Stöckhert, Postmagmatic cooling and late cenozoic denudation of the north patagonian batholith in the los lagos region of chile, 41°42° 15 s. Int. J. Earth Sci. 95, 504–528 (2006).

[80] A. Castro, C. Rodriguez, C. Fernández, E. Aragón, M. F. Pereira, J. F. Molina, Secular variations of magma source compositions in the North Patagonian batholith from the Jurassic to Tertiary: Was melange melting involved? Geosphere 17, 766–785 (2021).

[81] R. J. Pankhurst, S. D. Weaver, F. Hervé, P. Larrondo, Mesozoic-cenozoic evolution of the North Patagonian batholith in aysen, southern chile. J. Geol. Soc. 156, 673–694 (1999).

[82] A. Castro, I. Moreno-Ventas, C. Fernández, G. Vujovich, G. Gallastegui, N. Heredia, R. D. Martino, R. Becchio, L. G. Corretgé, J. Díaz-Alvarado, P. Such, M. García-Arias, D. Y. Liu, Petrology and shrimp u-pb zircon geochronology of cordilleran granitoids of the Bariloche area, Argentina. J. South Am. Earth Sci. 32, 508–530 (2011).

[83] R. J. Pankhurst, F. Hervé, L. Rojas, J. Cembrano, Magmatism and tectonics in continental Chiloe, Chile (42–42 30′ s). Tectonophysics 205, 283–294 (1992).

[84] O. Urbina, “Geología de la cordillera Norpatagonica en el área del Río Palena, xi region de Aysen, Chile,” thesis, Universidad de Chile, Departamento de Geología (2001).

[85] SRGM-BRGM, “Carta metalogénica x region sur, Chile” (Servicio Nacional de Geología y Minería-Bureau de Recherches Géologiques et Minières, Informe Registrado IR-95-05, 1995), vol. 10.

[86] F. Hervé, Rejuvenecimiento de edades radiométricas en la zona de falla Liquiñe-Ofqui, en Aysen. Comunicaciones 34, 103 (1984).

[87] C. Rapela, F. Munizaga, L. Dalla Salda, F. Herve, M. A. Parada, C. Cingolani, “Nuevas edades k-ar de los granitoides del sector nororiental de los Andes Patagonicos” (Congreso Geológico Argentino, 1987), vol. 4, pp. 18–20.

[88] E. F. Gonzalez-Díaz, Chronological zonation of granitic plutonism in the northern Patagonian Andes of Argentina: The migration of intrusive cycles. Earth Sci. Rev. 18, 365–393 (1982).

[89] E. G. Sepulveda, R. L. M. Viera, Geología y áreas de alteración en el Cerro Colorado y alrededores, Chubut noroccidental. Rev. Asoc. Geol. Argent 35, 195–202 (1980).

[90] M. Halpern, P. Stipanicic, R. Toubes, Geocronología (rb/sr) en los Andes Australes Argentinos. Rev. Asoc. Geol. Argent 30, 180–192 (1975).

[91] M. Halpern, R. Fuenzalida, Rubidium-strontium geochronology of a transect of the Chilean Andes between latitudes 45 and 46 s. Earth Planet. Sci. Lett. 41, 60–66 (1978).

[92] R. O. Toubes, J. P. Spikermann, Algunas edades k/ar y rb/sr de plutonitas de la cordillera Patagonica entre los paralelos 40 y 44 de latitud sur. Rev. Asoc. Geol. Argent 28, 382–396 (1973).

[93] A. H. Pesce, “Estratigrafía de la cordillera patagónica entre los paralelos 43°30′ y 44° de latitud sur y sus áreas mineralizadas, provincia de Chubut,” in *Actas del 7° Congreso Geológico Argentino* (Asociación Geológica Argentina, 1978), pp. 257–270.

[94] D. Stanzione, M. Barbieri, E. Godoy, M. J. Haller, M. R. Ghiara, C. Trudu, “Geoquímica del estroncio y de tierras raras en el batolito patagónico (43–46°S),” in *Actas del Congreso Geológico Chileno* (Congreso Geologico Chileno, 1991), vol. 1, pp. 679–683.

[95] J. C. Turner, “Descripción geológica de la Hoja 44c, Tecka, Provincia del Chubut” (Servicio Geológico Nacional, 1982).

[96] M. Barbieri, M. Ghiara, M. J. Haller, D. Stanzione, C. Trudu, Genesis and evolution of granitoids from the patagonian batholith between 43° and 46°s. Mineral Petrogr. Acta 37, 1–15 (1994).

[97] J. Spikermann, Contribución al conocimiento de la intrusividad en el paleozoico de la región extraandina del Chubut. Rev. Asoc. Geol. Argent 33, 17–35 (1978).

[98] P. J. Lesta, R. Ferello, “Región extraandina de Chubut y norte de Santa Cruz,” in *Geología Regional Argentina*, A. F. Leanza, Ed. (Academia Nacional de Ciencias, 1972), vol. 2, pp. 601–653.

[99] J. Benito, J. Chernicoff, Geología del Cerro Caquel y ledaños, departamento Futaleufú, Provincia del Chubut. Rev. Asoc. Geol. Argent 41, 2 (1986).

[100] V. A. Ramos, Descripción geológica de la Hoja 47 ab, lago Fontana, Provincia del Chubut: Carta geológica-económica de la República Argentina, escala 1:200.000 (Servicio Geológico Nacional, 1981).

[101] A. P. Rolando, L. A. Hartmann, J. O. S. Santos, R. R. Fernandez, R. O. Etcheverry, I. A. Schalamuk, N. J. McNaughton, Shrimp zircon U-Pb evidence for extended Mesozoic magmatism in the Patagonian batholith and assimilation of Archean crustal components. J. South Am. Earth Sci. 15, 267–283 (2002).

[102] M. Suárez, R. De la Cruz, Jurassic to Miocene K-Ar dates from eastern central Patagonian Cordillera plutons (45°-48°S). Geol. Mag. 138, 53–66 (2001).

[103] R. J. Pankhurst, T. R. Riley, C. M. Fanning, S. P. Kelley, Episodic silicic volcanism in Patagonia and the Antarctic Peninsula: Chronology of magmatism associated with the break-up of Gondwana. J. Petrol. 41, 605–625 (2000).

[104] K. Butler, “Mesozoic-Cenozoic broken foreland basin evolution in Northern Patagonia, Argentina (∼42-46°S): Integrating sedimentation, magmatism, and subduction dynamics,” thesis, University of Texas at Austin (2022).

[105] M. Martin, R. J. Pankhurst, C. M. Fanning, S. N. Thomson, M. Calderon, F. Herve, “Age distribution of plutons across the southern Patagonian batholith: New U-Pb data on zircons,” in *III South American Symposium on Isotope Geology* (Servicio Nacional de Geología y Minería, 2001), pp. 585–588.

[106] R. De la Cruz, J. Cortés, “Geología del área oriental de la Hoja Puerto Cisnes, Región Aysén del General Carlos Ibánez del Campo” (Servicio Nacional de Geología y Minería, Carta Geológica de Chile, Serie Geología Básica 127, 2011).

[107] M. Suárez, R. De La Cruz, M. C. Bell, “Geología del área Nireguao-Baño Nuevo, Región Aisén del General Carlos Ibánez del Campo” (Servicio Nacional de Geología y Minería, Carta Geológica de Chile, Serie Geología Básica, 2007).

[108] R. De la Cruz, M. Suárez, M. Belmar, D. Quiroz, M. Bell, “Área Coihaique-Balmaceda, Región de Aisén del General Carlos Ibánez del Campo” (Servicio Nacional de Geología y Minería, Carta Geológica de Chile, Serie Geología Básica 80, 2003).

[109] B. E. Boltshauser, C. B. Zaffarana, G. Gallastegui, D. L. Orts, J. F. Molina, S. M. N. Poma, V. R. González, Petrogenetic evolution and thermobarometry of the late Jurassic La Hoya pluton, early stages of the North Patagonian batholith, southwestern Argentina. Int. J. Earth Sci. 112, 1687–1716 (2023).

[110] M. A. Parada, A. Lahsen, C. Palacios, “Magmatic evolution of the eastern part of the Chilean Patagonia (Aysén region): Geochronological and geochemical constraints,” in *Géodynamique Andine: Résumé Étendus = Andean Geodynamics: Extended Abstracts* (Institut de Recherche pour le Développement/BRGM, 1996), pp. 617–620.

[111] M. A. Parada, C. Palacios, A. Lahsen, Jurassic extensional tectono-magmatism and associated mineralization of the El Faldeo polymetallic district, Chilean Patagonia: Geochemical and isotopic evidence of crustal contribution. Miner. Deposita 32, 547–554 (1997).

[112] R. De la Cruz, M. Suárez, “Geología del área Puerto Guadal-Puerto Sánchez, Región Aisén del General Carlos Ibánez del Campo” (Servicio Nacional de Geología y Minería, Carta Geológica de Chile, Serie Geológica Básica 95, 2006).

[113] D. Quiroz, Z. Bruce, “Geología del Área Puerto Ingeniero Ibañez-Villa Cerro Castillo, Región Aisén del General Carlos Ibánez del Campo” (Servicio Nacional de Geología y Minería, Carta Geológica de Chile, Serie Geología Básica 124, 2010).

[114] D. Quiroz, M. Belmar, “Geología del área Bahia Murta: Cerro sin Nombre, Región de Aisén del General Carlos Ibañez del Campo” (Servicio Nacional de Geología y Minería, Carta Geológica de Chile, Serie Geología Básica 125, 2010).

[115] M. E. Ramos, A. Folguera, L. Fennell, M. Giménez, V. D. Litvak, Y. Dzierma, V. A. Ramos, Tectonic evolution of the North Patagonian Andes from field and gravity data (39-40°S). J. South Am. Earth Sci. 51, 59–75 (2014).

[116] J. R. Franzese, L. D’Elia, A. Bilmes, M. Muravchik, M. Hernández, Superposición de cuencas extensionales y contraccionales Oligo-miocenas en el retroarco andino norpatagónico: La cuenca de Aluminé, Neuquén, Argentina. Andean Geol. 38, 319–334 (2011).

[117] C. W. Rapela, L. A. Spalletti, J. C. Merodio, E. Aragon, Temporal evolution and spatial variation of early tertiary volcanism in the Patagonian Andes (40°S-42°30’S). J. South Am. Earth Sci. 1, 75–88 (1988).

[118] C. W. Rapela, L. A. Spalletti, J. C. Merodio, Evolución magmática y geotectónica de la serie andesítica andina (Paleoceno-Eoceno) en la Cordillera Nordpatagónica. Rev. la Asoc. Geol. Argent. 38, 469–484 (1983).

[119] S. B. Iannelli, V. D. Litvak, L. Fernández Paz, A. Folguera, M. E. Ramos, V. A. Ramos, Evolution of Eocene to Oligocene arc-related volcanism in the North Patagonian Andes (39-41°S), prior to the break-up of the Farallon plate. Tectonophysics 696-697, 70–87 (2017).

[120] F. Bechis, A. Encinas, A. Concheyro, V. D. Litvak, B. Aguirre-Urreta, V. A. Ramos, New age constraints for the Cenozoic marine transgressions of northwestern Patagonia, Argentina (41-43°S): Paleogeographic and tectonic implications. J. South Am. Earth Sci. 52, 72–93 (2014).

[121] L. Fernández Paz, F. Bechis, V. D. Litvak, A. Echaurren, A. Encinas, J. González, F. Lucassen, V. Oliveros, V. Valencia, A. Folguera, Constraints on trenchward arc migration and backarc magmatism in the North Patagonian Andes in the context of Nazca plate rollback. Tectonics 38, 3794–3817 (2019).

[122] M. M. Mazzoni, K. Kawashita, S. Harrison, E. Aragón, Edades radimétricas eocenas. Borde occidental del macizo Norpatagónico. Rev. Asoc. Geol. Argent. 46, 150–158 (1991).

[123] L. Fernández Paz, S. B. Iannelli, A. Echaurren, M. Ramos, F. Bechis, V. D. Litvak, A. Encinas, S. Kasemann, F. Lucassen, A. Folguera, The late Eocene-early Miocene El Maitén belt evolution: Magmatic response to the changing subduction zone geodynamics. J. South Am. Earth Sci. 103, 102713 (2020).

[124] E. Aragón, A. Castro, J. Díaz-Alvarado, D.-Y. Liu, The North Patagonian batholith at Paso Puyehue (Argentina-Chile): Shrimp ages and compositional features. J. South Am. Earth Sci. 32, 547–554 (2011).

[125] L. Benedini, M. C. Geraldes, D. A. Gregori, L. Strazzere, P. Marcos, M. V. Barros, “Nueva edad U-Pb eocena tardía para la formación Ventana, Andes nordpatagónicos, provincia de Río Negro,” in *XX Congreso Geológico Argentino* (Asociación Geológica Argentina, 2017), pp. 7–11.

[126] R. E. Giacosa, N. C. Heredia, “Hoja geológica 4172-IV, San Carlos de Bariloche, Provincias de Río Negro y Neuquén” (Servicio Geológico Minero Argentino, 2002).

[127] A. Lizuaín, R. M. Viera, “Descripción geológica de la Hoja 4372-I y II, Esquel, Provincia de Chubut” (Servicio Geológico Minero Argentino, 2010).

