# Supplementary material for: Slab underthrusting is the primary control on flat-slab size
Source: Sci Adv. 2025 Jul 11;11(28):eadv8872. doi: 10.1126/sciadv.adv8872 (PMC12248310; doi:10.1126/sciadv.adv8872)
Supplement: Supplementary file 1 — Figs. S1 to S4 Tables S1 and S2 Legends for movies S1 to S3 Legends for data S1 and S2 References [file sciadv.adv8872_sm.pdf]

Science Advances
AAAS

Supplementary Materials for

Slab underthrusting is the primary control on flat-slab size

Guido M. Gianni *et al.*

Corresponding author: Guido M. Gianni, guidogianni22@gmail.com

Sci. Adv. **11**, eadv8872 (2025)
DOI: 10.1126/sciadv.adv8872

The PDF file includes:

Figs. S1 to S4
Tables S1 and S2
Legends for movies S1 to S3
Legends for data S1 and S2
References

Other Supplementary Material for this manuscript includes the following:

Movies S1 to S3
Data S1 and S2

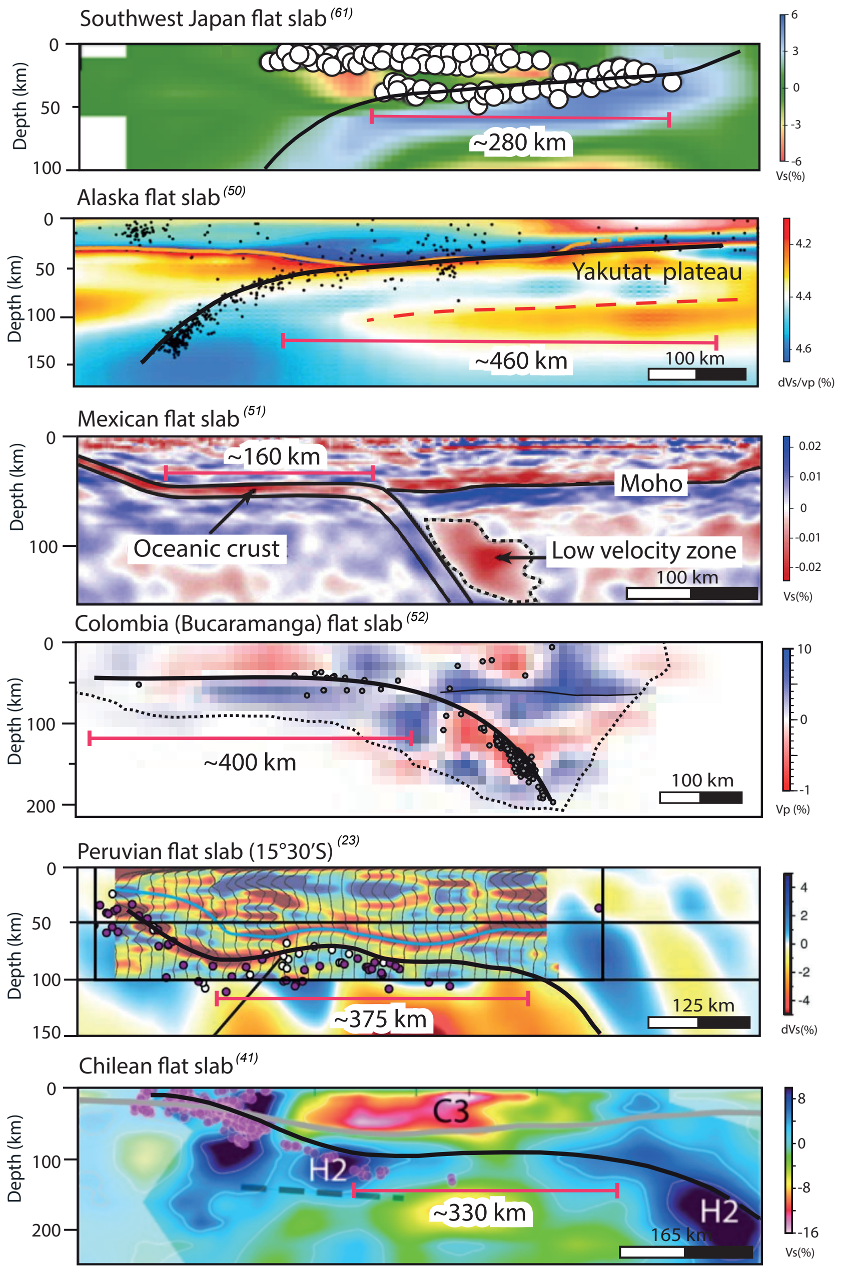

Figure S1: **Compilation of regional seismic tomography and/or receiver function at active flat slab locations**. Measured active flat slab lengths derived from the compiled seismological data were compared with the Slab2 subduction zone geometry model *(34)* to corroborate its resolution and suitability for the analysis presented in Fig. 2. In seismic tomography from the Chilean flat slab, H2 and C3 labels correspond to Nazca flat slab high velocity anomalies and crustal low velocity anomaly, respectively

.

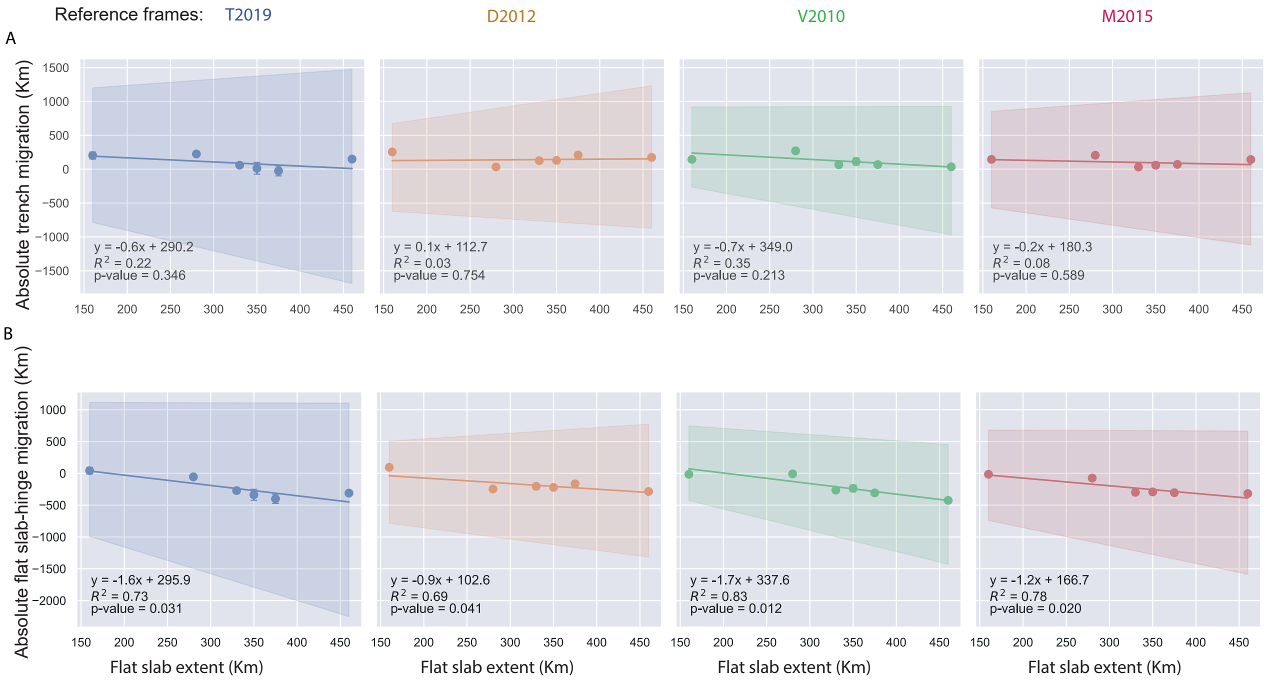

Figure S2: **Statistical analysis of the absolute trench and flat slab hinge motion versus flat subduction extents in active flat subduction settings**. Correlation analysis between **A** determined absolute trench and **B** flat slab hinge motion vs. flat slab extents derived from the Slab2 subduction geometry model *(34)*.

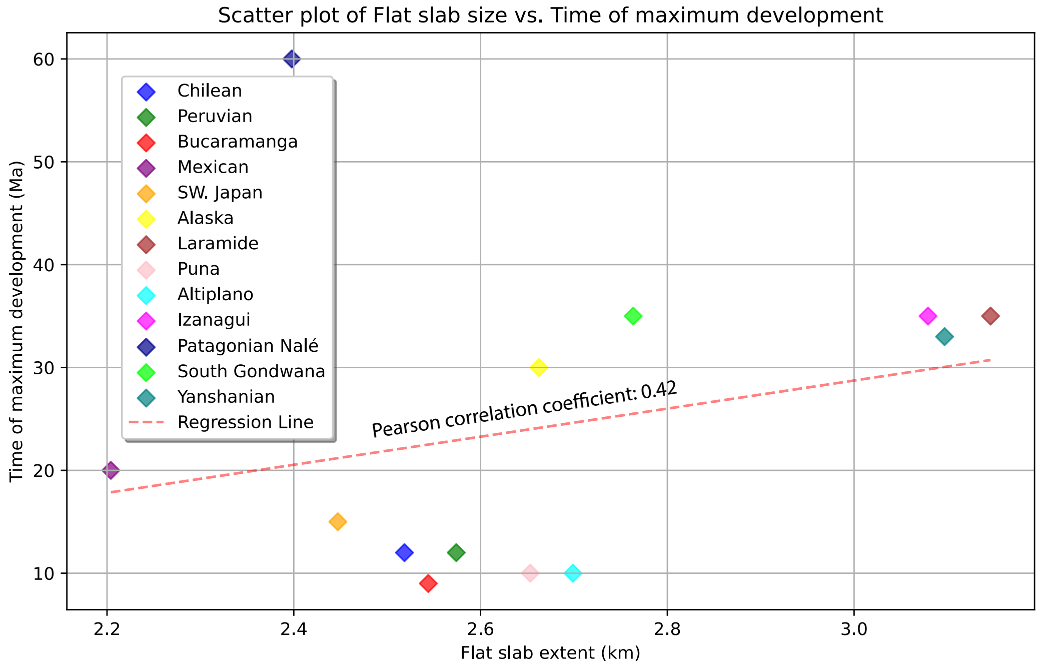

Figure S3: **Analysis of flat slab sizes vs. Time to maximum flat slab development**. In this diagram, a conservative kinked flat slab geometry is considered, which shows a moderate and positive correlation with the time to maximum flat slab development.

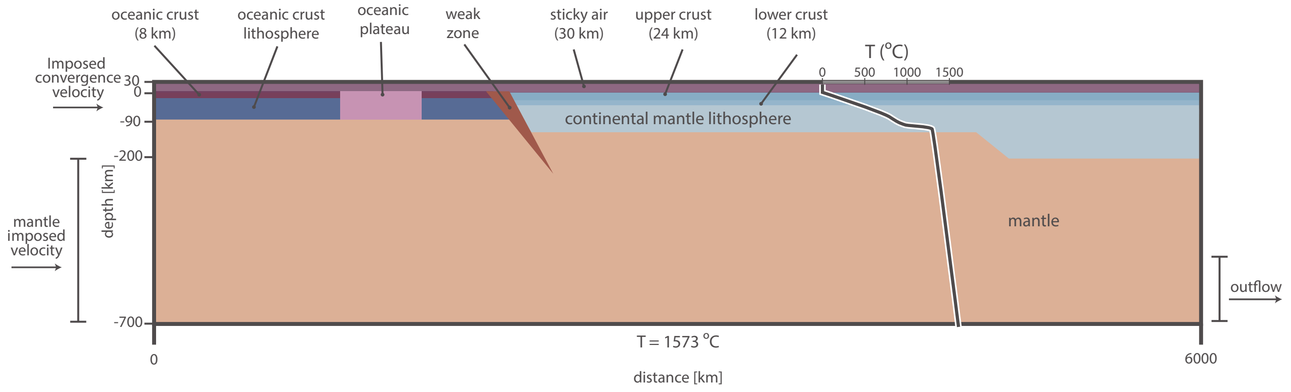

Figure S4: **2-D Geodynamic modeling Set-up of flat subduction formation and propagation.**

Table S1: Material Properties and model parameters.

	Oceanic crust	Oceanic crust lithosphere	Continental upper crust	Continental lower crust	Continental mantle lithosphere	Mantle	
Viscous rheology							
Material	Wet Olivine	Wet olivine	Wet quartzite	Dry Maryland diabase	Wet olivine	Dry olivine Diffusion	Dry olivine Dislocation
Aps (Pa^{-n} s^{-1})	$5.04{\times}10^{-28}$	$1.76{\times}10^{-14}$	$8.57{\times}10^{-28}$	$5.04{\times}10^{-28}$	$1.76{\times}10^{-14}$	1.5	1.1
n	4.7	3	4	4.7	3	3.5	1
E (kJ mol^{-1})	484	430	223	485	430	520	375
V* (cm^3 mol^{-1})	0	10	0	0	10	6	2.3
Density							
ρ (kg m^{-3})	2950	3200	2800	2950	3200	3250	
α (K^{-1})	$3.0x10^{-5}$	$3.0x10^{-5}$	$3.0x10^{-5}$	$3.0x10^{-5}$	$3.0x10^{-5}$	3.0x10-5	
Plastic rheology							
Co (MPa)	10	10	20	20	20	10	
Cohesion after softening (MPa)	2	2	3	3	3	10	
Friction coefficient	0.577	0.577	0.44	0.44	0.44	0.6	
Friction coefficient after softening	0.1154	0.1154	0.088	0.088	0.088	0.6	
Strain range of softening	0.0-2.0	0.0-2.0	0.0-0.5	0.0-0.5	0.0-0.5	1.5	

Table S2: **Model parameters of partial melting.**

Latent heat of fusion (kJ/kg)	250
Solidus coefficient ‘as’ (k)	1000
Solidus coefficient ‘bs’ (k/Pa)	-1.2e^{-7}
Solidus coefficient ‘cs’ (k/Pa2)	0.5e^{-16}
Liquidus coefficient ‘al’ (k)	1493
Liquidus coefficient ‘bl’ (k/Pa)	-1.2e^{-7}
Liquidus coefficient ‘cl’ (k/Pa2)	1.6e^{-16}
Viscous softening melt fraction	0.15-0.30

Caption for Movie S1. Video showing plastic strain in model A testing the role of fast mantle flow under low convergence on flat subduction forward propagation. Animated version of Figure 6A.

Caption for Movie S2. Video showing plastic strain in model B testing the role of an imposed average plate convergence rate on the lower plate on flat subduction forward propagation. Animated version of Figure 6B.

Caption for Movie S3. Video showing plastic strain in model C testing the role of slab pull on the forward propagation of flat subduction. Animated version of Figure 6C.

Supplementary Data S1. Flat slab data base and plate kinematic data used for statistical analysis of active and ancient flat slab cases.

This dataset includes the names and states of examined flat slab cases (‘name‘ and ‘state‘), derived flat slab extents from the Slab2 model and arc migration evolution following Methodologies A [flat_slab_size_min (A)] and B [flat_slab_size_max (B)]; both methods are detailed in the main text. It also includes the absolute reference frames used (‘reference_frame‘) [T2019, (*28,32*), V2010 (*30*), M2015 (*31*), and D2012 (*33, 37*)], average trench motion (‘trench_motion_mean‘) and standard deviations (‘trench_motion_std‘) along flat slab segments, and absolute flat slab hinge motion following Methods A and B [flat_slab_hinge_motion_(A)/(B)]. Additionally, the dataset provides the contributions of forward propagation of the flat slab hinge [contribution_flat_slab_hinge_(A)/(B)] and backward propagation [contribution_trench_(A)/(B)*] for each case, flat slab lifespans, and associated references, including flat slab onset and termination ages (for ancient cases).

Supplementary Data S2. Radiometric database for the Patagonian Nalé flat slab. Compilation of radiometric ages of igneous rocks corresponding to the Mesozoic-Early Cenozoic magmatic arc in Patagonia (*79–127*), used to track the formation and demise of the Cretaceous Nalé flat slab. This data was assembled to estimate the potential size of this flat slab segment.

REFERENCES AND NOTES

1. M. Barazangi, B. L. Isacks, Spatial distribution of earthquakes and subduction of the Nazca plate beneath South America. *Geology* **4**, 686–692 (1976).

2. B. L. Isacks, M. Barazangi, "Geometry of Benioff zones: Lateral segmentation and downwards bending of the subducted lithosphere," in *Island Arcs, Deep Sea Trenches and Back-Arc Basins* (John Wiley & Sons, 1977), vol. 1, pp. 99–114 .

3. P. J. Coney, S. J. Reynolds, Cordilleran benioff zones. *Nature* **270**, 403–406 (1977).

4. G. M. Gianni, S. P. Luján, Geodynamic controls on magmatic arc migration and quiescence. *Earth Sci. Rev.* **218**, 103676 (2021).

5. M.-A. Gutscher, W. Spakman, H. Bijwaard, E. R. Engdahl, Geodynamics of flat subduction: Seismicity and tomographic constraints from the Andean margin. *Tectonics* **19**, 814–833 (2000).

6. V. A. Ramos, E. O. Cristallini, D. J. Pérez, The pampean flat-slab of the central andes. *J. South Am. Earth Sci.* **15**, 59–78 (2002).

7. J. Martinod, M. Gérault, L. Husson, V. Regard, Widening of the Andes: An interplay between subduction dynamics and crustal wedge tectonics. *Earth Sci. Rev.* **204**, 103170 (2020).

8. G. M. Gianni, F. M. Dávila, A. Echaurren, L. Fennell, J. Tobal, C. Navarrete, P. Quezada, A. Folguera, M. Giménez, A geodynamic model linking Cretaceous orogeny, arc migration, foreland dynamic subsidence and marine ingression in southern South America. *Earth Sci. Rev.* **185**, 437–462 (2018).

9. L. Liu, D. Peng, L. Liu, L. Chen, S. Li, Y. Wang, Z. Cao, M. Feng, East Asian lithospheric evolution dictated by multistage Mesozoic flat-slab subduction. *Earth Sci. Rev.* **217**, 103621 (2021).

10. L. Liu, L. Liu, Y.-G. Xu, Mesozoic intraplate tectonism of East Asia due to flat subduction of a composite terrane slab. *Earth Sci. Rev.* **214**, 103505 (2021).

11. F.-Y. Wu, J.-H. Yang, Y.-G. Xu, S. A. Wilde, R. J. Walker, Destruction of the North China craton in the Mesozoic. *Annu. Rev. Earth Planet. Sci.* **47**, 173–195 (2019).

12. Z.-X. Li, X.-H. Li, Formation of the 1300-km-wide intracontinental orogen and postorogenic magmatic province in Mesozoic South China: A flat-slab subduction model. *Geology* **35**, 179–182 (2007).

13. D. Peng, L. Liu, Y. Wang, A newly discovered Late-Cretaceous East Asian flat slab explains its unique lithospheric structure and tectonics. *J. Geophys. Res. Solid Earth* **126**, e2021JB022103 (2021).

14. V. A. Ramos, A. Folguera, Andean flat-slab subduction through time. *Geol. Soc. Lond. Spec. Publ.* **327**, 31–54 (2009).

15. A. Folguera, V. A. Ramos, Repeated eastward shifts of arc magmatism in the Southern Andes: A revision to the long-term pattern of Andean uplift and magmatism. *J. South Am. Earth Sci.* **32**, 531–546 (2011).

16. J. van Hunen, A. P. van den Berg, N. J. Vlaar, The impact of the South-American plate motion and the Nazca Ridge subduction on the flat subduction below South Peru. *Geophys. Res. Lett.* **29**, 35-1–35-4 (2002).

17. J. van Hunen, A. P. van den Berg, N. J. Vlaar, Various mechanisms to induce present-day shallow flat subduction and implications for the younger Earth: A numerical parameter study. *Phys. Earth Planet. In.* **146**, 179–194 (2004).

18. N. Espurt, F. Funiciello, J. Martinod, B. Guillaume, V. Regard, C. Faccenna, S. Brusset, Flat subduction dynamics and deformation of the South American plate: Insights from analog modeling. *Tectonics* **27**, TC3011 (2008).

19. J. Martinod, B. Guillaume, N. Espurt, C. Faccenna, F. Funiciello, V. Regard, Effect of aseismic ridge subduction on slab geometry and overriding plate deformation: Insights from analogue modeling. *Tectonophysics* **588**, 39–55 (2013).

20. V. C. Manea, M. Pérez-Gussinyé, M. Manea, Chilean flat slab subduction controlled by overriding plate thickness and trench rollback. *Geology* **40**, 35–38 (2012).

21. J. Hu, L. Liu, A. Hermosillo, Q. Zhou, Simulation of late Cenozoic South American flat-slab subduction using geodynamic models with data assimilation. *Earth Planet. Sci. Lett.* **438**, 1–13 (2016).

22. G. M. Gianni, C. Navarrete, S. Spagnotto, Surface and mantle records reveal an ancient slab tear beneath Gondwana. *Sci. Rep.* **9**, 19774 (2019).

23. B. T. Bishop, S. L. Beck, G. Zandt, L. S. Wagner, M. D. Long, H. Tavera, Foreland uplift during flat subduction: Insights from the Peruvian Andes and Fitzcarrald Arch. *Tectonophysics* **731-732**, 73–84 (2018).

24. P. Copeland, C. A. Currie, T. F. Lawton, M. A. Murphy, Location, location, location: The variable lifespan of the Laramide orogeny. *Geology* **45**, 223–226 (2017).

25. S. Liu, C. A. Currie, Farallon plate dynamics prior to the Laramide orogeny: Numerical models of flat subduction. *Tectonophysics* **666**, 33–47 (2016).

26. G. Schepers, D. J. J. van Hinsbergen, W. Spakman, M. E. Kosters, L. M. Boschman, N. McQuarrie, South-American plate advance and forced Andean trench retreat as drivers for transient flat subduction episodes. *Nat. Commun.* **8**, 15249 (2017).

27. M. Barazangi, B. L. Isacks, Subduction of the Nazca plate beneath Peru: Evidence from spatial distribution of earthquakes. *Geophys. J. Int.* **57**, 537–555 (1979).

28. R. D. Müller, S. Zahirovic, S. E. Williams, J. Cannon, M. Seton, D. J. Bower, M. G. Tetley, C. Heine, E. le Breton, S. Liu, S. H. J. Russell, T. Yang, J. Leonard, M. Gurnis, A global plate model including lithospheric deformation along major rifts and orogens since the Triassic. *Tectonics* **38**, 1884–1907 (2019).

29. M. Seton, R. D. Müller, S. Zahirovic, C. Gaina, T. Torsvik, G. Shephard, A. Talsma, M. Gurnis, M. Turner, S. Maus, M. Chandler, Global continental and ocean basin reconstructions since 200 Ma. *Earth Sci. Rev.* **113**, 212–270 (2012).

30. D. G. van Der Meer, W. Spakman, D. J. Van Hinsbergen, M. L. Amaru, T. H. Torsvik, Towards absolute plate motions constrained by lower-mantle slab remnants. *Nat. Geosci.* **3**, 36–40 (2010).

31. S. Maher, P. Wessel, R. Müller, S. Williams, Y. Harada, Absolute plate motion of Africa around Hawaii-Emperor bend time. *Geophys. J. Int.* **201**, 1743–1764 (2015).

32. M. G. Tetley, S. E. Williams, M. Gurnis, N. Flament, R. D. Müller, Constraining absolute plate motions since the Triassic. *J. Geophys. Res. Solid Earth* **124**, 7231–7258 (2019).

33. P. V. Doubrovine, B. Steinberger, T. H. Torsvik, Absolute plate motions in a reference frame defined by moving hot spots in the Pacific, Atlantic, and Indian oceans. *J. Geophys. Res. Solid Earth* **117**, B09101 (2012).

34. G. P. Hayes, G. L. Moore, D. E. Portner, M. Hearne, H. Flamme, M. Furtney, G. M. Smoczyk, Slab2, a comprehensive subduction zone geometry model. *Science* **362**, 58–61 (2018).

35. C. Navarrete, G. Gianni, A. Encinas, M. Márquez, Y. Kamerbeek, M. Valle, A. Folguera, Triassic to Middle Jurassic geodynamic evolution of southwestern Gondwana: From a large flat-slab to mantle plume suction in a rollback subduction setting. *Earth Sci. Rev.* **194**, 125–159 (2019).

36. Y. Liu, L. Liu, Z. Wu, W. Li, X. Hao, New insight into East Asian tectonism since the late Mesozoic inferred from erratic inversions of NW-trending faulting within the Bohai Bay Basin. *Gondw. Res.* **102**, 17–30 (2022).

37. K. J. Matthews, K. T. Maloney, S. Zahirovic, S. E. Williams, M. Seton, R. D. Müller, Global plate boundary evolution and kinematics since the late Paleozoic. *Global Planet. Change* **146**, 226–250 (2016).

38. C. Montes, A. F. Rodriguez-Corcho, G. Bayona, N. Hoyos, S. Zapata, A. Cardona, Continental margin response to multiple arc-continent collisions: The northern Andes-Caribbean margin. *Earth Sci. Rev.* **198**, 102903 (2019).

39. X. Liu, L. S. Wagner, C. A. Currie, M. J. Caddick, Implications of flat-slab subduction on hydration, slab seismicity, and arc volcanism in the Pampean region of Chile and Argentina. *Geochem. Geophys. Geosyst.* **25**, e2023GC011317 (2024).

40. M. A. Jadamec, M. I. Billen, S. M. Roeske, Three-dimensional numerical models of flat slab subduction and the Denali fault driving deformation in south-central Alaska. *Earth Planet. Sci. Lett.* **376**, 29–42 (2013).

41. Y. Gao, X. Yuan, B. Heit, F. Tilmann, D. P. van Herwaarden, S. Thrastarson, A. Fichtner, B. Schurr, Impact of the Juan Fernandez Ridge on the Pampean flat subduction inferred from full waveform inversion. *Geophys. Res. Lett.* **48**, e2021GL095509 (2021).

42. D. Stevenson, J. Turner, Angle of subduction. *Nature* **270**, 334–336 (1977).

43. J. Rodríguez-González, A. M. Negredo, M. I. Billen, The role of the overriding plate thermal state on slab dip variability and on the occurrence of flat subduction. *Geochem. Geophys. Geosyst.* **13**, Q01002 (2012).

44. J. van Hunen, A. P. van den Berg, N. J. Vlaar, A thermo-mechanical model of horizontal subduction below an overriding plate. *Earth Planet. Sci. Lett.* **182**, 157–169 (2000).

45. D. B. Rowley, A. M. Forte, C. J. Rowan, P. Glišović, R. Moucha, S. P. Grand, N. A. Simmons, Kinematics and dynamics of the East Pacific Rise linked to a stable, deep-mantle upwelling. *Sci. Adv.* **2**, e1601107 (2016).

46. D. Forsyth, S. Uyeda, On the relative importance of the driving forces of plate motion. *Geophys. J. Int.* **43**, 163–200 (1975).

47. J. Rodríguez-González, A. M. Negredo, E. Carminati, Slab-mantle flow interaction: Influence on subduction dynamics and duration. *Terra Nova* **26**, 265–272 (2014).

48. P. Huangfu, Y. Wang, P. A. Cawood, Z. H. Li, W. Fan, T. V. Gerya, Thermo-mechanical controls of flat subduction: Insights from numerical modeling. *Gondw. Res.* **40**, 170–183 (2016).

49. S. Zahirovic, R. D. Müller, M. Seton, N. Flament, Tectonic speed limits from plate kinematic reconstructions. *Earth Planet. Sci. Lett.* **418**, 40–52 (2015).

50. R. Martin-Short, R. Allen, I. D. Bastow, R. W. Porritt, M. S. Miller, Seismic imaging of the Alaska subduction zone: Implications for slab geometry and volcanism. *Geochem. Geophys. Geosyst.* **19**, 4541–4560 (2018).

51. Y. Kim, R. W. Clayton, J. M. Jackson, Geometry and seismic properties of the subducting Cocos plate in central Mexico. *J. Geophys. Res.* **115**, B06310 (2010).

52. C. Chiarabba, P. de Gori, C. Faccenna, F. Speranza, D. Seccia, V. Dionicio, G. A. Prieto, Subduction system and flat slab beneath the Eastern Cordillera of Colombia. *Geochem. Geophys. Geosyst.* **17**, 16–27 (2016).

53. D. J. van Hinsbergen, W. Spakman, H. de Boorder, M. van Dongen, S. M. Jowitt, P. R. D. Mason, Arc-type magmatism due to continental-edge plowing through ancient subduction-enriched mantle. *Geophys. Res. Lett.* **47**, e2020GL087484 (2020).

54. R. González, O. Oncken, C. Faccenna, E. le Breton, M. Bezada, A. Mora, Kinematics and convergent tectonics of the Northwestern South American plate during the Cenozoic. *Geochem. Geophys. Geosyst.* **24**, e2022GC010827 (2023).

55. L. Wagner, J. S. Jaramillo, L. F. Ramírez-Hoyos, G. Monsalve, A. Cardona, T. W. Becker, Transient slab flattening beneath Colombia. *Geophys. Res. Lett.* **44**, 6616–6623 (2017).

56. V. C. Manea, M. Manea, L. Ferrari, T. Orozco-Esquivel, R. W. Valenzuela, A. Husker, V. Kostoglodov, A review of the geodynamic evolution of flat slab subduction in Mexico, Peru, and Chile. *Tectonophysics* **695**, 27–52 (2017).

57. E. J. Moreno, V. C. Manea, M. Manea, S. Yoshioka, N. Suenaga, A. Bayona, Numerical modeling of subduction and evaluation of Philippine Sea Plate tectonic history along the Nankai Trough. *Sci. Rep.* **13**, 18313 (2023).

58. J. M. Trop, J. A. Benowitz, C. S. Kirby, M. E. Brueseke, Geochronology of the Wrangell Arc: Spatial-temporal evolution of slab-edge magmatism along a flat-slab, subduction-transform transition, Alaska-Yukon. *Geosphere* **18**, 19–48 (2022).

59. T. Waldien, R. O. Lease, S. Roeske, J. Benowitz, P. O'Sullivan, The role of preexisting upper plate strike-slip faults during long-lived (ca. 30 Myr) oblique flat slab subduction, southern Alaska. *Earth Planet. Sci. Lett.* **577**, 117242 (2022).

60. T. H. Torsvik, L. R. M. Cocks, The integration of palaeomagnetism, the geological record and mantle tomography in the location of ancient continents. *Geol. Mag.* **156**, 242–260 (2019).

61. K. Asamori, D. Zhao, Teleseismic shear wave tomography of the Japan subduction zone. *Geophys. J. Int.* **203**, 1752–1772 (2015).

62. E. S. Finzel, J. M. Trop, K. D. Ridgway, E. Enkelmann, Upper plate proxies for flat-slab subduction processes in southern Alaska. *Earth Planet. Sci. Lett.* **303**, 348–360 (2011).

63. L. Liu, M. Gurnis, M. Seton, J. Saleeby, R. D. Müller, J. M. Jackson, The role of oceanic plateau subduction in the Laramide orogeny. *Nat. Geosci.* **3**, 353–357 (2010).

64. E. Scheuber, K.-J. Reutter, Magmatic arc tectonics in the Central Andes between 21 and 25 S. *Tectonophysics* **205**, 127–140 (1992).

65. T. Habel, M. Simoes, R. Lacassin, D. Carrizo, G. Aguilar, A contribution to the quantification of crustal shortening and kinematics of deformation across the Western Andes (~ 20–22° S). *Solid Earth* **14**, 17–42 (2023).

66. B. Steinberger, T. H. Torsvik, Absolute plate motions and true polar wander in the absence of hotspot tracks. *Nature* **452**, 620–623 (2008).

67. C. O'Neill, D. Müller, B. Steinberger, On the uncertainties in hot spot reconstructions and the significance of moving hot spot reference frames. *Geochem. Geophys. Geosyst.* **6**, Q04003 (2005).

68. B. Steinberger, R. Sutherland, R. J. O'connell, Prediction of Emperor-Hawaii seamount locations from a revised model of global plate motion and mantle flow. *Nature* **430**, 167–173 (2004).

69. T. H. Torsvik, R. D. Müller, R. Van der Voo, B. Steinberger, C. Gaina, Global plate motion frames: Toward a unified model. *Rev. Geophys.* **46**, RG3004 (2008).

70. R. Beucher, L. Moresi, J. Giordani, J. Mansour, D. Sandiford, R. Farrington, L. Mondy, C. Mallard, P. Rey, G. Duclaux, O. Kaluza, A. Laik, S. Morón, UWGeodynamics: A teaching and research tool for numerical geodynamic modelling. *J. Open Source Softw.* **4**, 1136 (2019).

71. L. Moresi, F. Dufour, H.-B. Mühlhaus, A Lagrangian integration point finite element method for large deformation modeling of viscoelastic geomaterials. *J. Comput. Phys.* **184**, 476–497 (2003).

72. L. Moresi, S. Quenette, V. Lemiale, C. Mériaux, B. Appelbe, H. B. Mühlhaus, Computational approaches to studying non-linear dynamics of the crust and mantle. *Phys. Earth Planet. Inter.* **163**, 69–82 (2007).

73. C. Rosenberg, M. Handy, Experimental deformation of partially melted granite revisited: Implications for the continental crust. *J. Metam. Geol.* **23**, 19–28 (2005).

74. C. Beaumont, M. Nguyen, R. A. Jamieson, S. Ellis, Crustal flow modes in large hot orogens. *Geol. Soc. Spec. Publ.* **268**, 91–145 (2006).

75. G. C. Gleason, J. Tullis, A flow law for dislocation creep of quartz aggregates determined with the molten salt cell. *Tectonophysics* **247**, 1–23 (1995).

76. S. Mackwell, M. Zimmerman, D. Kohlstedt, High-temperature deformation of dry diabase with application to tectonics on Venus. *J. Geophys. Res. Solid Earth* **103**, 975–984 (1998).

77. S.-I. Karato, P. Wu, Rheology of the upper mantle: A synthesis. *Science* **260**, 771–778 (1993).

78. G. M. Gianni, L. Gallo, Jupyter Notebooks and files to accompany the paper: Slab underthrusting is the primary control on flat slab size, Zenodo (2025); https://doi.org/10.5281/zenodo.15558004.

79. A. C. Adriasola, S. N. Thomson, M. R. Brix, F. Hervé, B. Stöckhert, Postmagmatic cooling and late cenozoic denudation of the north patagonian batholith in the los lagos region of chile, 41°42° 15 s. *Int. J. Earth Sci.* **95**, 504–528 (2006).

80. A. Castro, C. Rodriguez, C. Fernández, E. Aragón, M. F. Pereira, J. F. Molina, Secular variations of magma source compositions in the North Patagonian batholith from the Jurassic to Tertiary: Was melange melting involved? *Geosphere* **17**, 766–785 (2021).

81. R. J. Pankhurst, S. D. Weaver, F. Hervé, P. Larrondo, Mesozoic-cenozoic evolution of the North Patagonian batholith in aysen, southern chile. *J. Geol. Soc.* **156**, 673–694 (1999).

82. A. Castro, I. Moreno-Ventas, C. Fernández, G. Vujovich, G. Gallastegui, N. Heredia, R. D. Martino, R. Becchio, L. G. Corretgé, J. Díaz-Alvarado, P. Such, M. García-Arias, D. Y. Liu, Petrology and shrimp u-pb zircon geochronology of cordilleran granitoids of the Bariloche area, Argentina. *J. South Am. Earth Sci.* **32**, 508–530 (2011).

83. R. J. Pankhurst, F. Hervé, L. Rojas, J. Cembrano, Magmatism and tectonics in continental Chiloe, Chile (42–42 30′ s). *Tectonophysics* **205**, 283–294 (1992).

84. O. Urbina, “Geología de la cordillera Norpatagonica en el área del Río Palena, xi region de Aysen, Chile,” thesis, Universidad de Chile, Departamento de Geología (2001).

85. SRGM-BRGM, “Carta metalogénica x region sur, Chile” (Servicio Nacional de Geología y Minería-Bureau de Recherches Géologiques et Minières, Informe Registrado IR-95-05, 1995), vol. 10.

86. F. Hervé, Rejuvenecimiento de edades radiométricas en la zona de falla Liquiñe-Ofqui, en Aysen. *Comunicaciones* **34**, 103 (1984).

87. C. Rapela, F. Munizaga, L. Dalla Salda, F. Herve, M. A. Parada, C. Cingolani, "Nuevas edades k-ar de los granitoides del sector nororiental de los Andes Patagonicos" (Congreso Geológico Argentino, 1987), vol. 4, pp. 18–20.

88. E. F. Gonzalez-Díaz, Chronological zonation of granitic plutonism in the northern Patagonian Andes of Argentina: The migration of intrusive cycles. *Earth Sci. Rev.* **18**, 365–393 (1982).

89. E. G. Sepulveda, R. L. M. Viera, Geología y áreas de alteración en el Cerro Colorado y alrededores, Chubut noroccidental. *Rev. Asoc. Geol. Argent* **35**, 195–202 (1980).

90. M. Halpern, P. Stipanicic, R. Toubes, Geocronología (rb/sr) en los Andes Australes Argentinos. *Rev. Asoc. Geol. Argent* **30**, 180–192 (1975).

91. M. Halpern, R. Fuenzalida, Rubidium-strontium geochronology of a transect of the Chilean Andes between latitudes 45 and 46 s. *Earth Planet. Sci. Lett.* **41**, 60–66 (1978).

92. R. O. Toubes, J. P. Spikermann, Algunas edades k/ar y rb/sr de plutonitas de la cordillera Patagonica entre los paralelos 40 y 44 de latitud sur. *Rev. Asoc. Geol. Argent* **28**, 382–396 (1973).

93. A. H. Pesce, "Estratigrafía de la cordillera patagónica entre los paralelos 43°30′ y 44° de latitud sur y sus áreas mineralizadas, provincia de Chubut," in *Actas del 7° Congreso Geológico Argentino* (Asociación Geológica Argentina, 1978), pp. 257–270.

94. D. Stanzione, M. Barbieri, E. Godoy, M. J. Haller, M. R. Ghiara, C. Trudu, "Geoquímica del estroncio y de tierras raras en el batolito patagónico (43–46°S)," in *Actas del Congreso Geológico Chileno* (Congreso Geologico Chileno, 1991), vol. 1, pp. 679–683.

95. J. C. Turner, "Descripción geológica de la Hoja 44c, Tecka, Provincia del Chubut" (Servicio Geológico Nacional, 1982).

96. M. Barbieri, M. Ghiara, M. J. Haller, D. Stanzione, C. Trudu, Genesis and evolution of granitoids from the patagonian batholith between 43° and 46°s. *Mineral Petrogr. Acta* **37**, 1–15 (1994).

97. J. Spikermann, Contribución al conocimiento de la intrusividad en el paleozoico de la región extraandina del Chubut. *Rev. Asoc. Geol. Argent* **33**, 17–35 (1978).

98. P. J. Lesta, R. Ferello, "Región extraandina de Chubut y norte de Santa Cruz," in *Geología Regional Argentina*, A. F. Leanza, Ed. (Academia Nacional de Ciencias, 1972), vol. 2, pp. 601–653.

99. J. Benito, J. Chernicoff, Geología del Cerro Caquel y ledaños, departamento Futaleufú, Provincia del Chubut. *Rev. Asoc. Geol. Argent* **41**, 2 (1986).

100. V. A. Ramos, Descripción geológica de la Hoja 47 ab, lago Fontana, Provincia del Chubut: Carta geológica-económica de la República Argentina, escala 1:200.000 (Servicio Geológico Nacional, 1981).

101. A. P. Rolando, L. A. Hartmann, J. O. S. Santos, R. R. Fernandez, R. O. Etcheverry, I. A. Schalamuk, N. J. McNaughton, Shrimp zircon U-Pb evidence for extended Mesozoic magmatism in the Patagonian batholith and assimilation of Archean crustal components. *J. South Am. Earth Sci.* **15**, 267–283 (2002).

102. M. Suárez, R. De la Cruz, Jurassic to Miocene K-Ar dates from eastern central Patagonian Cordillera plutons (45°-48°S). *Geol. Mag.* **138**, 53–66 (2001).

103. R. J. Pankhurst, T. R. Riley, C. M. Fanning, S. P. Kelley, Episodic silicic volcanism in Patagonia and the Antarctic Peninsula: Chronology of magmatism associated with the break-up of Gondwana. *J. Petrol.* **41**, 605–625 (2000).

104. K. Butler, "Mesozoic-Cenozoic broken foreland basin evolution in Northern Patagonia, Argentina (~42-46°S): Integrating sedimentation, magmatism, and subduction dynamics," thesis, University of Texas at Austin (2022).

105. M. Martin, R. J. Pankhurst, C. M. Fanning, S. N. Thomson, M. Calderon, F. Herve, "Age distribution of plutons across the southern Patagonian batholith: New U-Pb data on zircons," in *III South American Symposium on Isotope Geology* (Servicio Nacional de Geología y Minería, 2001), pp. 585–588.

106. R. De la Cruz, J. Cortés, “Geología del área oriental de la Hoja Puerto Cisnes, Región Aysén del General Carlos Ibánez del Campo” (Servicio Nacional de Geología y Minería, Carta Geológica de Chile, Serie Geología Básica 127, 2011).

107. M. Suárez, R. De La Cruz, M. C. Bell, “Geología del área Nireguao-Baño Nuevo, Región Aisén del General Carlos Ibánez del Campo” (Servicio Nacional de Geología y Minería, Carta Geológica de Chile, Serie Geología Básica, 2007).

108. R. De la Cruz, M. Suárez, M. Belmar, D. Quiroz, M. Bell, “Área Coihaique-Balmaceda, Región de Aisén del General Carlos Ibánez del Campo” (Servicio Nacional de Geología y Minería, Carta Geológica de Chile, Serie Geología Básica 80, 2003).

109. B. E. Boltshauser, C. B. Zaffarana, G. Gallastegui, D. L. Orts, J. F. Molina, S. M. N. Poma, V. R. González, Petrogenetic evolution and thermobarometry of the late Jurassic La Hoya pluton, early stages of the North Patagonian batholith, southwestern Argentina. *Int. J. Earth Sci.* **112**, 1687–1716 (2023).

110. M. A. Parada, A. Lahsen, C. Palacios, “Magmatic evolution of the eastern part of the Chilean Patagonia (Aysén region): Geochronological and geochemical constraints,” in *Géodynamique Andine: Résumé Étendus = Andean Geodynamics: Extended Abstracts* (Institut de Recherche pour le Développement/BRGM, 1996), pp. 617–620.

111. M. A. Parada, C. Palacios, A. Lahsen, Jurassic extensional tectono-magmatism and associated mineralization of the El Faldeo polymetallic district, Chilean Patagonia: Geochemical and isotopic evidence of crustal contribution. *Miner. Deposita* **32**, 547–554 (1997).

112. R. De la Cruz, M. Suárez, “Geología del área Puerto Guadal-Puerto Sánchez, Región Aisén del General Carlos Ibánez del Campo” (Servicio Nacional de Geología y Minería, Carta Geológica de Chile, Serie Geológica Básica 95, 2006).

113. D. Quiroz, Z. Bruce, “Geología del Área Puerto Ingeniero Ibañez-Villa Cerro Castillo, Región Aisén del General Carlos Ibánez del Campo” (Servicio Nacional de Geología y Minería, Carta Geológica de Chile, Serie Geología Básica 124, 2010).

114. D. Quiroz, M. Belmar, "Geología del área Bahia Murta: Cerro sin Nombre, Región de Aisén del General Carlos Ibañez del Campo" (Servicio Nacional de Geología y Minería, Carta Geológica de Chile, Serie Geología Básica 125, 2010).

115. M. E. Ramos, A. Folguera, L. Fennell, M. Giménez, V. D. Litvak, Y. Dzierma, V. A. Ramos, Tectonic evolution of the North Patagonian Andes from field and gravity data (39-40°S). *J. South Am. Earth Sci.* **51**, 59–75 (2014).

116. J. R. Franzese, L. D'Elia, A. Bilmes, M. Muravchik, M. Hernández, Superposición de cuencas extensionales y contraccionales Oligo-miocenas en el retroarco andino norpatagónico: La cuenca de Aluminé, Neuquén, Argentina. *Andean Geol.* **38**, 319–334 (2011).

117. C. W. Rapela, L. A. Spalletti, J. C. Merodio, E. Aragon, Temporal evolution and spatial variation of early tertiary volcanism in the Patagonian Andes (40°S-42°30'S). *J. South Am. Earth Sci.* **1**, 75–88 (1988).

118. C. W. Rapela, L. A. Spalletti, J. C. Merodio, Evolución magmática y geotectónica de la serie andesítica andina (Paleoceno-Eoceno) en la Cordillera Nordpatagónica. *Rev. la Asoc. Geol. Argent.* **38**, 469–484 (1983).

119. S. B. Iannelli, V. D. Litvak, L. Fernández Paz, A. Folguera, M. E. Ramos, V. A. Ramos, Evolution of Eocene to Oligocene arc-related volcanism in the North Patagonian Andes (39-41°S), prior to the break-up of the Farallon plate. *Tectonophysics* **696-697**, 70–87 (2017).

120. F. Bechis, A. Encinas, A. Concheyro, V. D. Litvak, B. Aguirre-Urreta, V. A. Ramos, New age constraints for the Cenozoic marine transgressions of northwestern Patagonia, Argentina (41-43°S): Paleogeographic and tectonic implications. *J. South Am. Earth Sci.* **52**, 72–93 (2014).

121. L. Fernández Paz, F. Bechis, V. D. Litvak, A. Echaurren, A. Encinas, J. González, F. Lucassen, V. Oliveros, V. Valencia, A. Folguera, Constraints on trenchward arc migration and backarc magmatism in the North Patagonian Andes in the context of Nazca plate rollback. *Tectonics* **38**, 3794–3817 (2019).

122. M. M. Mazzoni, K. Kawashita, S. Harrison, E. Aragón, Edades radimétricas eocenas. Borde occidental del macizo Norpatagónico. *Rev. Asoc. Geol. Argent.* **46**, 150–158 (1991).

123. L. Fernández Paz, S. B. Iannelli, A. Echaurren, M. Ramos, F. Bechis, V. D. Litvak, A. Encinas, S. Kasemann, F. Lucassen, A. Folguera, The late Eocene-early Miocene El Maitén belt evolution: Magmatic response to the changing subduction zone geodynamics. *J. South Am. Earth Sci.* **103**, 102713 (2020).

124. E. Aragón, A. Castro, J. Díaz-Alvarado, D.-Y. Liu, The North Patagonian batholith at Paso Puyehue (Argentina-Chile): Shrimp ages and compositional features. *J. South Am. Earth Sci.* **32**, 547–554 (2011).

125. L. Benedini, M. C. Geraldes, D. A. Gregori, L. Strazzere, P. Marcos, M. V. Barros, "Nueva edad U-Pb eocena tardía para la formación Ventana, Andes nordpatagónicos, provincia de Río Negro," in *XX Congreso Geológico Argentino* (Asociación Geológica Argentina, 2017), pp. 7–11.

126. R. E. Giacosa, N. C. Heredia, "Hoja geológica 4172-IV, San Carlos de Bariloche, Provincias de Río Negro y Neuquén" (Servicio Geológico Minero Argentino, 2002).

127. A. Lizuaín, R. M. Viera, "Descripción geológica de la Hoja 4372-I y II, Esquel, Provincia de Chubut" (Servicio Geológico Minero Argentino, 2010).
